# Behaviour change interventions to improve physical activity in adults: a systematic review of economic evaluations

**DOI:** 10.1186/s12966-024-01614-6

**Published:** 2024-07-09

**Authors:** Stephen Barrett, Stephen Begg, Jack Lawrence, Gabrielle Barrett, Josh Nitschke, Paul O’Halloran, Jeff Breckon, Marina De Barros Pinheiro, Catherine Sherrington, Chris Doran, Michael Kingsley

**Affiliations:** 1 Research and Innovation, Bendigo Health, Barnard St, Victoria, VIC 3552 Australia; 2 Holsworth Research Initiative, La Trobe University, Bendigo, VIC 3552 Australia; 3 Violet Vines Marshman Centre for Rural Health Research, La Trobe Rural Health School, Bendigo, VIC Australia; 4 Outpatient Rehabilitation, Bendigo Health, Victoria, 3552 Australia; 5 Centre for Sport and Social Impact, La Trobe University, Melbourne, 3086 Australia; 6 School of Psychology and Public Health, La Trobe University, Melbourne, 3086 Australia; 7 School of Health & Life Sciences, Teesside University, Middlesbrough, TS1 3BA North Yorkshire UK; 8 Institute for Musculoskeletal Health, The University of Sydney and Sydney Local Health District, Sydney, NSW 2006 Australia; 9 Sydney School of Public Health, Faculty of Medicine and Health, The University of Sydney, Sydney, NSW Australia; 10 Centre for Resilience and Wellbeing, Central Queensland University, Queensland, Australia; 11 Department of Exercise Sciences, University of Auckland, Auckland, New Zealand

## Abstract

**Background:**

Behaviour change interventions can result in lasting improvements in physical activity (PA). A broad implementation of behaviour change interventions are likely to be associated with considerable additional costs, and the evidence is unclear whether they represent good value for money. The aim of this study was to investigate costs and cost-effectiveness of behaviour change interventions to increase PA in community-dwelling adults.

**Methods:**

A search for trial-based economic evaluations investigating behaviour change interventions versus usual care or alternative intervention for adults living in the community was conducted (September 2023). Studies that reported intervention costs and incremental cost-effectiveness ratios (ICERs) for PA or quality-adjusted life years (QALYs) were included. Methodological quality was assessed using the Consensus Health Economic Criteria (CHEC-list). A Grading of Recommendations Assessment, Development and Evaluation style approach was used to assess the certainty of evidence (low, moderate or high certainty).

**Results:**

Sixteen studies were included using a variety of economic perspectives. The behaviour change interventions were heterogeneous with 62% of interventions being informed by a theoretical framework. The median CHEC-list score was 15 (range 11 to 19). Median intervention cost was US$313 per person (range US$83 to US$1,298). In 75% of studies the interventions were reported as cost-effective for changes in PA (moderate certainty of evidence). For cost per QALY/gained, 45% of the interventions were found to be cost-effective (moderate certainty of evidence). No specific type of behaviour change intervention was found to be more effective.

**Conclusions:**

There is moderate certainty that behaviour change interventions are cost-effective approaches for increasing PA. The heterogeneity in economic perspectives, intervention costs and measurement should be considered when interpreting results. There is a need for increased clarity when reporting the functional components of behaviour change interventions, as well as the costs to implement them.

**Supplementary Information:**

The online version contains supplementary material available at 10.1186/s12966-024-01614-6.

## Introduction

Despite the unequivocal benefits of regular physical activity (PA), physical inactivity remains a worldwide issue that causes substantial ill-health and subsequent related economic burdens [1]. Increasing PA is a health priority that is crucial in the prevention of several non-communicable diseases [2]. The high prevalence of physical inactivity has resulted in the widespread interest in the design and delivery of interventions to increase PA [3].

Structured exercise interventions have been a frontline strategy for addressing physical inactivity and sedentary lifestyle behaviours. Nevertheless, once structured interventions finish, the majority of individuals do not maintain the behaviour change [4]. As a result there has been an increasing interest in the use of behaviour change interventions to increase and maintain PA.

Behaviour change interventions incorporate different strategies and behaviour change techniques to promote change. These can include, but are not limited to increasing self-efficacy, self-regulation skills and capacity for maintenance [5]. Evidence from a number of reviews indicates that behaviour change interventions can result in lasting improvements in PA [3, 5–7]. The broad implementation of such interventions is likely to be associated with considerable additional costs, compared to single initial activities. Due to the limited resources available, policy makers need to be informed of interventions that provide best value for money [8]. Therefore, evaluations of behaviour change interventions aiming to increase PA should include both effectiveness and cost-effectiveness.

Intervention fidelity plays an important role in the cost-effectiveness of interventions by ensuring that resources are used efficiently, outcomes are maximised, and results are reliable [9]. Consistent fidelity in intervention delivery can reduce variability in outcomes across different settings or implementation sites [10], and is important to consider when interpreting overall value for money. Behaviour change interventions delivered with high fidelity can lead to greater improvement in outcomes [11], and may improve the overall value of interventions in terms of their impact on health and well-being.

Systematic reviews have previously been carried out to investigate the cost-effectiveness of PA interventions, but these reviews focused on structured exercise interventions [12], population health and community-level approaches [13–15] and placed-based approaches [16]. For example, Lutz and colleagues review examined the cost-effectiveness of behaviour change interventions in work places [16]. To the best of our knowledge, no systematic review has focused on the cost-effectiveness of behaviour change interventions to increase PA in adults.

The aim of this review was to summarise the evidence from economic evaluations and costing studies of behaviour change interventions to increase PA in adults free-living in the community. The review questions were: (1) what are the costs of developing and implementing behaviour change interventions to increase PA?; and (2) what is the cost-effectiveness of behaviour change interventions to increase PA?

## Methods

The Preferred Reporting Items for Systematic Reviews and Meta-Analyses statement (PRISMA checklist) and the guideline for recommendations for conducting systematic reviews of economic evaluations to inform evidence-based healthcare decisions were used in reporting this review [17–19]. The PRISMA checklist is provided in additional file 1. A protocol was prospectively registered and published on PROSPERO: CRD42022371485.

### Data sources

The following specialised databases were searched from inception to September 2023: Medline (Ovid), Embase (Ovid), CINAHL and PsycINFO. Searches were also carried out in the following registries: the National Institute for Health Research Economic Evaluation Database (NHS EED, via Centre for Reviews and Dissemination (CRD)), Research Papers in Economics (RePEc, via EconPapers) and EconLit (EBSCO). Search details for MEDLINE are presented in additional file 2. Finally, a hand search of the reference lists of the studies included in this review and other relevant systematic reviews was conducted.

### Eligibility

The following inclusion criteria were defined:

#### Population

Adults (≥ 18 years) free-living in the community. Consequently, studies where individuals were inpatients in healthcare facilities or residents in aged care facilities were excluded. Studies that examined worksite populations were also excluded; cost-effectiveness of interventions to increase physical activity in the workplace has already been examined [16].

#### Intervention

Any behaviour change intervention aimed to increase PA. For the purpose of this review, interventions that specifically aimed to elicit PA behaviour change through the use of behaviour change techniques (BCTs) were included [20, 21]. The behaviour change intervention(s) had to be delivered as a real-time intervention (i.e., asynchronous store-and-forward interventions were excluded). Multicomponent interventions [22] were included where the behaviour change intervention for PA constitutes a substantial component of the program (e.g., an intervention that included eight sessions of structured supervised exercise and one session of PA coaching would be excluded).

#### Comparator

Usual care, structured PA intervention, non-physical activity-related advice or no intervention.

#### Outcomes

For PA, the effectiveness was measured in changes of units of PA (e.g., minutes/day of PA). For quality of life, the effectiveness was measured in changes in quality-adjusted life-years (QALYs). Outcomes of interests needed to be presented in incremental cost effectiveness ratios (ICERs); the ICER represents the additional cost of one unit of outcome gained by one strategy compared with another. ICERs could be expressed as the incremental cost per change in PA, or the incremental cost per QALY gained. Outcomes of interests also included the cost of delivering the intervention.

#### Types of studies

Studies included in this reviews had to be clinical trial-based cost-effectiveness or cost-utility studies. The studies included randomised controlled trial and quasi-randomised trials. Studies that were not trial-based, for example model-based evaluations where data was derived from sources such as databases or extant literature, were excluded. Studies published in language other than English were excluded.

### Study selection and data extraction

Studies were entered into Covidence (Covidence Systematic Review Software, Veritas Health Innovation, Melbourne, Australia) and duplicates were removed. Two authors independently screened title/abstracts and full text. Studies were excluded when they did not meet the pre-specified inclusion criteria. Disagreements between reviewers were first resolved by discussion; where a decision was not reached a third reviewer was used to reach consensus.

Two independent reviewers extracted study information into a standardised form in Covidence. The following data were extracted: study design, study population, intervention and control group components including theoretical frameworks and measures of fidelity, outcome definition and measurement (device measured or self-reported PA), and results. Where information was not reported in the economic evaluation publication, data were extracted from additional publications relating to the same study, e.g., primary effectiveness manuscripts where the main trial results were reported or study protocols. When information was unclear, insufficient or missing, authors of trials were contacted for clarifications and additional results. The authors’ conclusion on the cost-effectiveness of interventions were extracted from included studies and are reported in this study.

### Methodological quality assessment

Methodological quality assessment was conducted using the Consensus on Health Economic Criteria list (CHEC-list) [23]. The CHEC-list consists of 19 yes or no questions and is suitable for evaluating trial-based economic evaluations [23]. Higher CHEC-list scores indicate higher methodological quality of the study. Two independent reviewers rated each study; a third reviewer was involved where disagreements occurred. In completing the CHEC-list, the information provided in the included study were considered, as well as relevant information from additional publications cited in the study such as primary effectiveness manuscripts where the main trial results were reported or study protocols.

### Strategy for assessing the certainty of evidence

A Grading of Recommendations Assessment, Development, and Evaluation (GRADE) style rating was used to assess the overall quality of the trial-based economic analyses. This GRADE style rating for economic reviews was created by Pinhiero and colleagues [24] and was based on the concepts identified in the GRADE approach [25]. The following domains were considered: (1) quality of trial-based reporting, (2) certainty of trial, (3) credibility of economic evaluation, (4) certainty of economic evaluation results, (5) applicability of trial. Each domain was rated as “poor”, “fair” or “good”. The overall certainty of each economic evaluation trial was rated as high, moderate, low, or very low by considering the ratings for the individual domains (additional files 3 and 4).

### Data synthesis

Standard deviations and 95% confidence intervals for cost data were often not reported in the economic evaluations, which made pooling costs difficult [16]. In addition, the time horizons and outcome measures differed substantially among the included studies. Due to this heterogeneity, pooling the results was considered to be inappropriate [26].

Several steps were undertaken to enhance the comparability of the included economic evaluations. In the included studies, the reported costs were provided in different currencies and costed from different years. The cost of the behaviour change interventions are expressed in two ways in this review: (1) by the year and currency reported in the included study, and (2) by price converted to 2021 $US to enable a comparison across studies. To convert the originally published prices the reported costs were inflated to 2021 costs using the inflation rate for each country from the Organisation for Economic Co-operation and Development (OECD) database [27]. Following this, the costs in respective currencies were transformed into US dollars using purchasing power parity conversion factors for 2021 [28].

To enhance comparability of PA results, where possible, the PA effect measures were standardised. The standardised PA measure used was the metabolic equivalent of task (MET) measured in MET-hours gained per person per day. One MET is defined as energy expenditure at rest and is equivalent to an oxygen consumption of 3.5 ml/kg/min [29]. The MET of an activity represents the intensity of an activity. The formula by Wu and colleagues [13] was used to transform PA outcomes to MET-hours gained per person per day. For these calculations, 3.0 METs were assigned to moderate PA, 4.5 METs to moderate-to-vigorous PA and 6.0 METs to vigorous PA. These values were chosen to be consistent with other studies in the field [12–14].

Willingness-to-pay thresholds are typically used to examine if an intervention is worthwhile i.e. the probability that the intervention is cost-effective at the price per unit increase [30]. If the ICER (cost per change in unit of PA or QALY/gained) is less than the willingness-to-pay thresholds, the indication is that funding the intervention may be a cost-effective strategy [30]. At present, there is no fixed willingness-to-pay threshold for PA change. To enhance comparability, a benchmark willingness-to-pay of $US0.50 to $US1.00 per MET-hour gained was used to examine the cost-effectiveness of included behaviour change interventions. This benchmark was based on the WHO recommendations for PA [13, 31] and has been applied in economic evaluations of PA interventions [12, 13]. To do this, mean differences in costs and outcomes between intervention and control were calculated to provide an estimation of ICERs in $US per MET-hour gained. Where interventions had different follow-up times the outcome in MET-hours per person per day was multiplied by the number of days of follow-up. Multiplying the MET-hours per person per day by the number of days of follow-up made the outcome comparable to the costs and allowed for comparison of interventions with different follow-up times.

Willingness-to-pay thresholds for QALYs gained often fall under the commonly used threshold of $US50 000 per QALYs gained proposed for medical treatments and procedures [32]. The threshold of $US50 000 per QALYs gained was used to examine the cost-effectiveness of included behaviour change interventions.

## Results

**Fig. 1:**
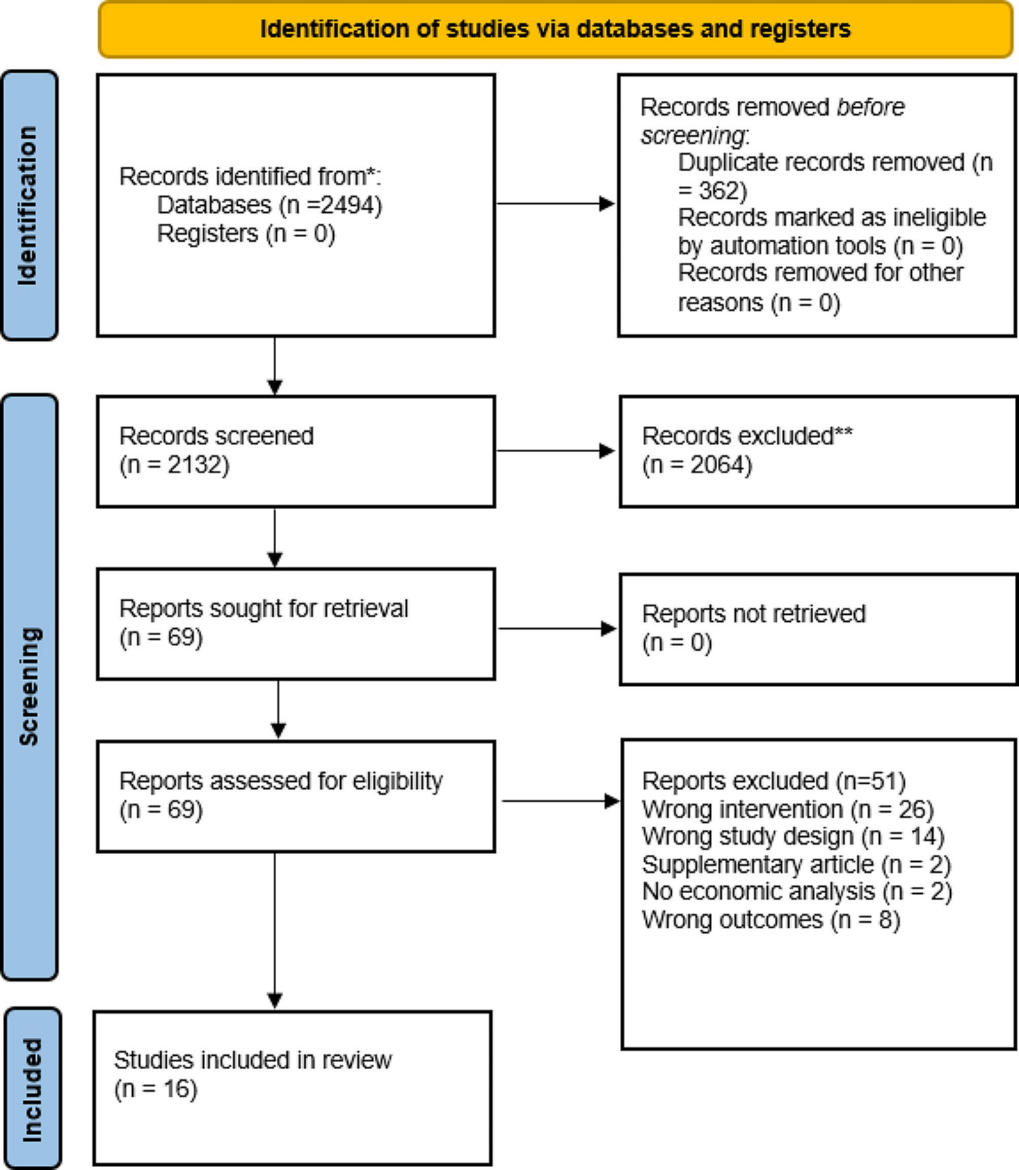
PRISMA flow diagram of study selection process

Following de-duplication, 2132 studies were screened. The PRISMA diagram for the screening is shown in Fig. 1. Sixteen full-text articles fulfilled the inclusion criteria and were included in the syntheses [33–48]. The studies excluded at full text review and the reason for exclusion are provided in additional file 5.

### Study characteristics

Characteristics of study populations and behaviour change interventions are detailed in Table 1. The economic characteristic of the studies including intervention costs are provided in Table 2. Fifteen studies were trial-based economic evaluations [33–38, 40–48], and one trial-based study included post-trial economic modelling [39]. All 16 studies were conducted in OECD member countries. Four studies (25%) were conducted in Australia [33, 34, 43, 47] and four (25%) the United States of America [37, 38, 44, 45].

**Table 1:** Characteristics of the included studies (*n* = 16)

Study	Country	Population	*N* (% females)	What was delivered	Who delivered it	Fidelity assessed	No of sessions	Intervention duration	Control group
Barrett, 2019	Australia	Insufficiently physically active adults recruited from ambulatory medical clinics	72 (75%)	Physical activity coaching using integrated MI-CBT	Allied health clinician	Yes	8	12 weeks	30-min group education session
Barrett, 2022	Australia	Insufficiently active adults recruited from ambulatory medical clinics	120 (68%)	Physical activity coaching using integrated MI-CBT	Physiotherapist	Yes	5	12 weeks	30-min group education session
Brodin, 2015	Sweden	Adults with rheumatic arthritis	228 (73%)	Physical activity coaching intervention	Physiotherapist	No	12	52 weeks	Ordinary physiotherapy treatment
Broekhuizen, 2018	European multisite *	Pregnant females	435 (100%)	Lifestyle coaching	Lifestyle coaches	No	5; 4 optional sessions	Tailored to the participants’ preference	Did not receive any of the lifestyle interventions.
Buder, 2018	USA	Females from minority cultural and ethnic groups	483 (100%)	Wellness-coaching program using motivational interviewing and personalised goal setting	Wellness coaches	No	12	4 weeks	In-person motivational interviewing wellness coaching 4, 8, and 12 months
Crist, 2022	USA	Adults 50 years and over	476 (76%)	Multilevel physical activity intervention including health coaching	Volunteer peer health coaches	No	NS	24 months	No intervention
Goyder, 2014	United Kingdom	Sedentary adults aged 40 to 64 years	282 (54%)	Physical activity consultations provided in a motivational interviewing style	Research assistants	Yes	2	8 weeks	Usual care
Ismail, 2020	United Kingdom	Adults aged 40 to 74 years with CVD risk	1742 (15%)	Motivational interviewing with additional behaviour change techniques	Health trainers	Yes	10 sessions	52 weeks	Usual care
Jacobs, 2010	Belgium	Highly educated adults aged between 25–75 years.	314 (33%)	Individual coaching based on Theory of Planned Behaviour and the Self-Determination Theory	Psychologist; assisted by undergraduate students in Sports and Nutrition	No	Determined by participant	Determined by participant	A preventive health consultation in general practice
Khunti, 2021	United Kingdom	Adults with risk of T2DM	1366 (49%)	A 3-hour group education and behaviour change program. Telephone calls at 1 week and 6 months. A group-based refresher session every year for 3 years	Nurse, dietitian or non-registered professionals (e.g. health trainer)	No	6	52 weeks	A 3-hour group education and behaviour change programme called Walking Away from Type 2 Diabetes. A group-based refresher session every year for 3 years
Sangster, 2015	Australia	People with cardiac disease	313 (27%)	Physical activity telephone-coaching based on social cognitive theory	Rehabilitation nurse	No	4	6 weeks	4 behavioural coaching and goal-setting sessions on weight, nutrition, and physical activity via telephone, plus 2 booster calls
Sevick, 2000	USA	Adults aged 35 years to 60 years who are overweight and insufficiently active	235 (NS)	Integrated behaviour modification and cognitive-behaviour modification techniques	Exercise psychologist, nutritionist and a health educator	No	34	26 weeks	Structured exercise intervention that included supervised, centre-based exercise
Sevick, 2007	USA	Healthy but sedentary adults ages 18 to 65	239 (82%)	Physical activity counselling based on Stages of Motivational Readiness for Change Model and Social Cognitive Theory.	Health educator	No	14	52 weeks	Education mail unrelated to physical activity
Sorensen, 2022	Denmark	Adults with rheumatoid arthritis	150 (80%)	Individual motivational counselling sessions and weekly individualised text messages	Healthcare professional	No	3	16 weeks	Participants were encouraged to maintain their usual lifestyle
Turkstra, 2013	Australia	Patients with a recent myocardial infarction	430 (25%)	Telephone health coaching sessions	Health professional or health coach	No	10	26 weeks	Received an existing written educational resource containing information about CVD and the associated risk factors
vanKeulen, 2010	Denmark	Adults aged 45 to 70 not meeting physical activity guidelines	1629 (45%)	Telephone motivational interviewing	Bachelor’s and master’s students of Health Education and Health Promotion, Mental Health Sciences or Psychology	Yes	4	43 weeks	Tailored print material

CVD: Cardiovascular disease; MI-CBT: Motivational interviewing and cognitive behaviour therapy; NS: Not stated; T2DM: Type 2 Diabetes Mellitus

* Countries included in multisite study: Austria, Belgium Denmark, Ireland, Italy, Netherlands, Poland, Spain, United Kingdom

**Table 2:** Economic characteristics of the included studies (*n* = 16)

Author, year	Economic evaluation	Perspective	Follow-up duration	Intervention costs per person $USD*	CHEC-list score	Study conclusions	Certainty of evidence
Barrett, 2019	CEA	Hospital perspective	26 weeks	$177	15	Telephone coaching was a low-cost strategy for increasing MVPA and QALYs in insufficiently physically active ambulatory care hospital patients.	Moderate
Barrett, 2022	CEA	Healthcare funder	39 weeks	$83	15	Physical activity telephone coaching was a low-cost strategy for increasing MVPA and QALYs in insufficiently active ambulatory hospital patients.	Moderate
Brodin, 2015	CEA	Societal perspective	52 weeks	$1028	15	Ordinary physiotherapy was most cost effective with regard to quality of life.	Moderate
Broekhuizen, 2018	CEA	Societal perspective	37 weeks	$598	15	The intervention was cost-effective for QALYs.	Moderate
Buder, 2018	CUA	NS	52 weeks	$751	11	This health coaching intervention was deemed to be cost-effective.	Low
Crist et al., 2022	CEA	Payer perspective	104 weeks	$302	13	This study provides evidence of a highly cost-effective intervention to increase PA and improve QALYs in older adults over a 2-year period.	Moderate
Goyder, 2014	CEA	NHS societal perspective	39 weeks	$324	19	The lack of impact on objectively measured physical activity levels suggest that it is unlikely to represent a clinically effective or cost-effective intervention.	High
Ismail, 2020	CEA	Healthcare funder	52 weeks	$184	15	The individual interventions was not cost-effective at conventional thresholds	Moderate
Jacobs, 2010	CUA	Healthcare funder	52 weeks	$825	17	The intervention was cost-effective after 1 year of intervention.	Moderate
Khunti, 2021	CEA	NHS perspective	Lifetime horizon	$446	18	The economic evaluation showed that the intervention was not cost effective for changes in physical activity.	Moderate
Sangster, 2015	CEA	Partial societal perspective	42 weeks	$145	16	The Healthy Weight intervention is overall both less costly and more effective compared to the Physical Activity coaching intervention.	Moderate
Sevick, 2000	CEA	Clinician perspective	102 weeks	$618	15	The intervention in which participants are taught behavioral skills to increase their physical activity was more cost-effective than a structured exercise program in improving physical activity.	Moderate
Sevick, 2007	CEA	Payer perspective	102 weeks	$1298	16	The intervention provides an efficient approach to increasing physical activity.	Moderate
Sorensen, 2022	CEA	Healthcare funder	96 weeks	$508	15	The individually tailored intervention is effective at improving participants’ health status and reducing healthcare costs.	Moderate
Turkstra, 2013	CEA	Healthcare funder	102 weeks	$212	13	There was no intervention effect measured and ProActive Heart resulted in significantly increased costs. The cost per QALYs gained was high and above acceptable limits compared to usual care.	Moderate
vanKeulen, 2010	CEA	Healthcare funder	78 weeks	$155	15	The control group displayed the most cost-efficacy for the number of QALYs experienced over 73 weeks	Moderate

CEA: Cost-effectiveness analysis; CUA: Cost-utility analysis; CVD: Cardiovascular disease; MVPA: moderate-to-vigorous physical activity; PA: Physical activity; QALYs: Quality-adjusted life years; WTP: Willingness-to-pay;

* Intervention costs represented in 2021 $US

^a^ costs converted from to 2021 $US

### Methodological quality of the evidence

The median CHEC-list score was 15 out of 19 (range 11 to 19) (additional file 6). General limitations in the included economic evaluations included: narrow perspectives for economic evaluations (not taking a societal perspectives) (*n* = 11, 69%), lack of exploration of uncertainty in sensitivity analysis (*n* = 5, 31%), and lack of reporting on potential conflict of interest of study researchers and funders (*n* = 9, 56%).

### Certainty of the evidence

Our GRADE style rating [24] demonstrated an overall moderate level of certainty of evidence. In 14 of the 16 (88%) included studies the level of certainty was moderate indicating that the outputs are likely to be reliable for decision making, but there is a possibility the outputs are not a reliable prediction of the cost-effectiveness. For one study (6%), confidence that the outputs from the economic evaluation are reliable for decision-making was rated as high [39]. For one study (6%), confidence that the outputs from the economic evaluation are reliable for decision-making was rated as low [37]. Rating summaries for all studies are provided in Table 2. Full details on GRADE style rating for included studies are provided in additional file 4.

### Behaviour change interventions

The behaviour change interventions delivered in the included studies varied. The median duration was 26 weeks (range 4 to 104 weeks). The median number of sessions delivered was 7 (range 2 to 34 session). The median follow-up duration was 52 weeks (range 26 to 104 weeks). The cost to deliver the intervention ranged from $US83 to $US1,298 per/person with a median cost of $US313 per/person. Intervention costs were predominantly provided as a total aggregated cost; the breakdown of the total cost to design and deliver the interventions were poorly reported. For example, the cost to train the persons delivering the intervention was only provided in two studies [34, 35] and costing data for provision of educational material were provided in three studies only [33, 34, 38].

In 10 of the 16 included studies the primary objective of the behaviour change intervention was changes in PA [33–35, 38, 39, 42–46]; in the other six studies the behaviour change intervention was focused on more than one outcome, for example changes in PA and diet [36, 37, 40, 41, 47, 48]. Physical activity was measured using accelerometers [33, 34, 38, 39, 42, 46] and questionnaires [35, 41, 44, 45, 47, 48]. Instruments used to calculate QALYs included (Short Form Health Survey) SF-36 [41, 47], SF-12 [33, 34, 39, 42], SF-6 [48], EQ-5D [35–37, 40, 42, 46] and the Assessment of Quality of Life 4D tool [43].

The description of the behaviour change interventions used in each study is provided in Table 1. The use of a theoretical framework to underpin the behaviour change intervention was described in 10 of the 16 (62%) studies. Four studies (25%) used motivational interviewing [37, 39, 40, 48], in two studies (12.5%) the intervention was integrated motivational interviewing and cognitive behaviour therapy (MI-CBT) [33, 34]. Other theoretical frameworks included social cognitive theory (12.5%) [43, 45] theory of planned behaviour and self-determination theory (6%) [45]. Other intervention descriptions not informed by theoretical frameworks included lifestyle coaching (6%) [36] health coaching (12.5%) [38, 47] or PA counselling (12.5%) [35, 45]. A measurement of intervention fidelity was reported in five of the 16 studies (31%); [33, 34, 39, 40, 48] in the five studies (31%) that measured fidelity the behaviour change intervention was underpinned by a theoretical framework. The comparators for all studies are detailed in Table 1.

### Economic evaluations

Data relating to cost-effectiveness analysis for changes in PA were available for eight studies [33–35, 38, 41, 45, 46, 48]. Overall, in six studies the authors reported that the behaviour change intervention was cost-effective for PA change; [33, 34, 38, 41, 45, 46] the level of certainty for all of these studies were moderate indicting that the results are likely to be reliable for decision making, but there is a possibility the outputs are not a reliable prediction of the cost-effectiveness of the intervention. In two studies the behaviour change intervention was more effective and less costly than the control and was considered the economically “dominant” strategy [33, 46]. For example, the ICER in the study by Barrett and colleagues [33] was -$AU61 per additional min of MVPA per day, meaning that $61 were saved per each additional minute of MVPA per day. In one study the intervention was more costly and less effective than the control group, therefore the intervention was ‘dominated’ by the control [48]. The level of certainty for this study was moderate [48].

Eight studies provided sufficient PA data to calculate the MET-hours gained and associated ICERs (Table 3) [33, 34, 38, 40, 41, 44, 45, 48]. The highest effect on changes in MET-hours gained was seen in an intervention using telephone delivered MI-CBT [34]. The gain of 0.98 MET-hours per day is equivalent to 20 min of moderate PA per day. Another intervention using integrated behaviour modification and cognitive-behaviour modification techniques yielded a gain of 0.84 MET-hours per day [44]. In total, four of the eight interventions resulted in gains of between 0.5 and 1 MET-hour per day [33, 34, 44, 45]. One intervention that used motivational interviewing with additional behaviour change techniques resulted in a negative effect (− 0.08 MET-hours per day) [40]. Accelerometers were used to measure PA in three of these eight studies [33, 34, 38], with average gains of 0.58 MET-hours per day in these studies. The average gains from studies using self-reported measured were 0.35 MET-hours per day.

**Table 3:** MET-hours gained and associated ICER of studies with physical activity data (*n* = 8)

Author, Year	Group	MET-hours gained per person per day	MET-hours gained per person	Intervention costs per person (US$ 2021)	Δ Effect (MET-hours gained)	Δ costs (US$ 2021)	ICERs per MET-hour gained (US$ 2021)
Barrett, 2019	C	-0.75	-136.5	13			
	I	0.98	177.45	177	313.9	164	0.52
Barrett, 2022	C	-0.3	-81.9	10			
	I	0.53	143.33	83	225.2	73	0.32
Crist, 2022	C	-0.3	-219	0			
	I	0.25	182.5	598	401.5	598	1.49
Ismail, 2020	C	-0.06	-46.53	0			
	I	-0.08	-62.05	184	15.52	184	11.8
Jacobs, 2010	C	0.15	57.35	208			
	I	0.17	62.57	825	5.21	617	118.3
Sevick, 2000	C	0.69	503.7	1032			
	I	0.84	613.2	359	109.5	-673	-6.1
Sevick, 2007	C	0.45	163.3	197			
	I	0.58	211.8	1299	48.5	1102	22.7
vanKeulen, 2010	C	0.25	125.9	0			
	I	0.26	131.4	157	5.5	157	28.5

C: Control group; I: Intervention group; Δ intervention − control group, ICER incremental cost-effectiveness ratio; MET-hours gained per person: MET-hours gained per person per day multiplied by the number of days of follow-up to make the effect comparable to the costs and, therefore, allow to compare interventions with different follow-up times

**Fig. 2:**
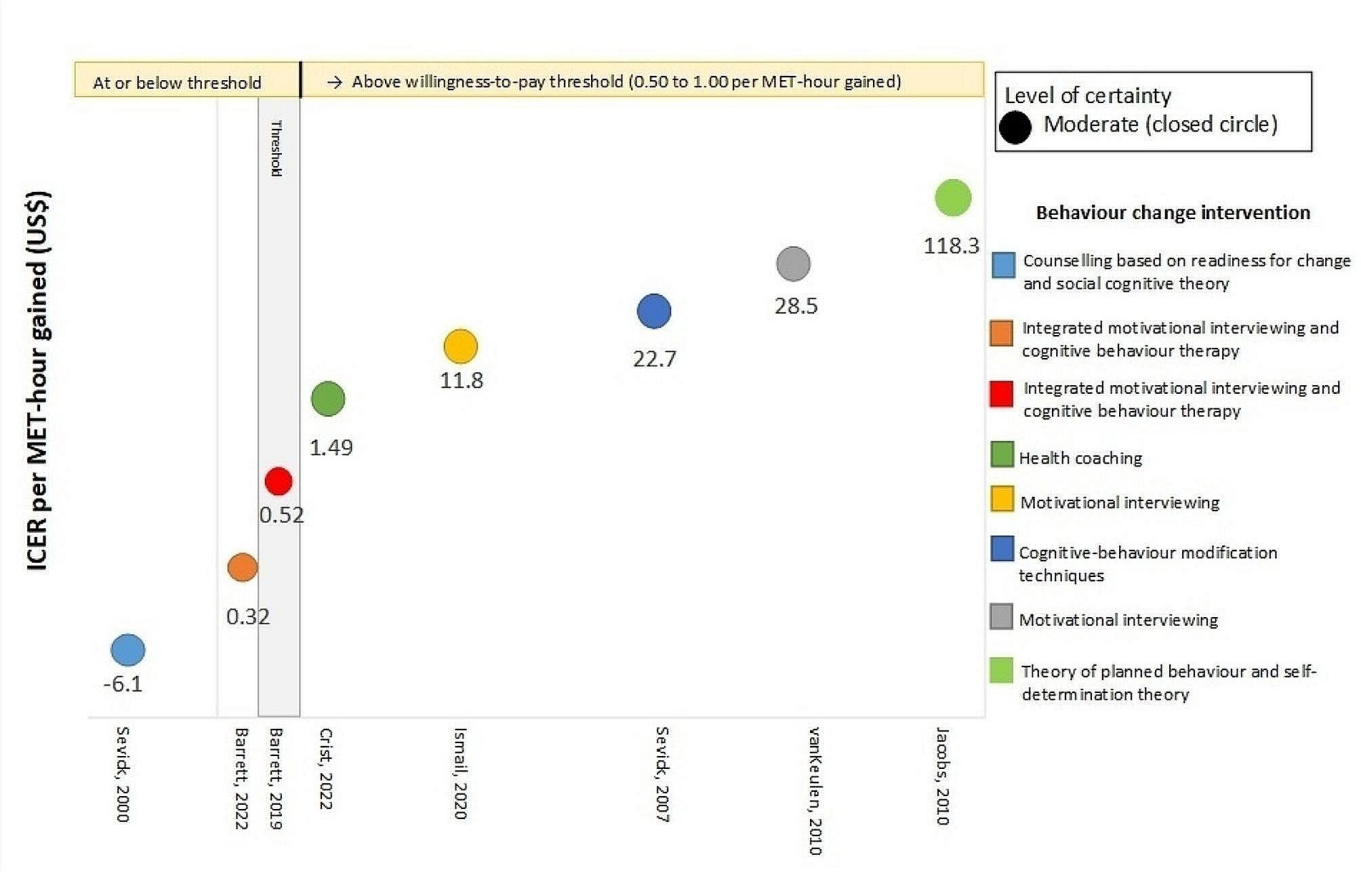
Incremental cost-effectiveness ratios (ICERs) expressed as additional cost per MET-hour gained (US$). The applied benchmark for cost-effectiveness of (S0.50 to 1.00 per MET-hour gained) is represented by the grey shaded area. Outcomes at or below threshold are deemed cost-effective at that willingness to pay. Closed circles indicate moderate level of certainty. Uncertainty intervals for ICERs are not displayed as most studies did not report it

The ICERs per MET-hour gained varied between the interventions (Fig. 2). Three interventions had an ICER within the applied benchmark of US$0.5–US$1.00 per MET-hour gained [33, 34, 44]. Two of these used telephone delivered MI-CBT, assessed PA with accelerometers and measured intervention fidelity [33, 34]. In both these studies the comparator was a single, low-cost group education session [33, 34]. In the other study with an ICER within the applied benchmark the PA counselling was compared to structured centre-based exercise [44]. The high costs to deliver the centre-based exercises (in this case the ‘control’) attributed to the difference in costs, and the associated ICER for the behaviour change intervention. The remaining five interventions had an ICER above the US$1.00 per MET-hour gained. The highest ICER reported was in a study with relatively high intervention costs and resulted in a modest change in MET-hours gained per person per day [41].

In total, 11 studies reported ICERs for QALYS gained. In five studies the behaviour change intervention was a deemed cost-effective strategy considering a willingness-to-pay of US50,000/QALY [33, 34, 36, 41, 46]. The behaviour change intervention was dominant (more effective and less costly) in three studies [33, 36, 46], and the cost per/QALYs gained for Barrett and colleagues [34] and Jacobs and colleagues [41] both fell under conventional willingness-to-pay thresholds. In these five studies, the comparator was a single group education session [33, 34], a preventive health consultation in general practice [41] or no intervention [36, 46]. In six of the 11 studies the behaviour change intervention was not deemed a cost-effective strategy for QALYs gained. In four studies the intervention was dominated (more costly and less effective) by control [39, 40, 43, 48]. The ICERs reported by Khunti and colleagues [42] and Turkstra and colleagues [47] both fell outside of conventional willingness-to-pay thresholds and were unlikely to be cost-effective. The level of certainty for all of these studies were moderate indicting that the results are likely to be reliable for decision making, but there is a possibility the outputs are not a reliable prediction of the cost-effectiveness of the intervention.

## Discussion

The results of this systematic review provide a comprehensive overview of trial-based economic evaluations of behaviour change interventions and detail evidence regarding the cost-effectiveness of behaviour change interventions designed to facilitate changes in PA. Intervention costs and ICERs were poorly reported. In particular, the differing measures of PA limited the ability to pool and directly compare results. Overall, results of the cost-effectiveness analyses varied widely. In six of eight included studies the interventions were reported as cost effective; three of the eight interventions were deemed cost-effective for improving PA when examined against an applied benchmark. The level of certainty these studies were moderate which indicated that the results are likely to be reliable for decision making. In regard to costs per QALYs/gained, the results varied and 5 of the 11 interventions would be cost-effective at to the willingness-to-pay threshold applied of 50,000/QALY gained. The level of certainty for these studies were moderate which indicated that the results of are likely to be reliable for decision making.

The heterogeneity in the behaviour change interventions used in the studies made direct comparisons difficult. Ten of the sixteen interventions were underpinned by a theoretical framework. Motivational interviewing was described as the framework for the intervention in three studies; in all three the intervention was not a cost-effective strategy to improve PA [39, 40, 48]. Tursktra and colleagues labelled their intervention ‘motivational counselling’, though their staff were trained in motivational interviewing principles and techniques [47]. This intervention was deemed cost-effective for improving PA [47]. Where motivational interviewing was integrated with cognitive behaviour therapy (MI-CBT) as a combined therapy these interventions were found to be cost-effective [33, 34]. Overall, the interventions were poorly described in many studies. Many of the included studies did not provide information on intervention content, schedule or measures of fidelity. Only five of the 16 included studies assessed the fidelity of the intervention; of the three interventions were deemed cost-effective for increasing PA, two measured intervention fidelity as part of the study [33, 34]. Without a clear measurement of fidelity, reports of the effectiveness of interventions need to be interpreted cautiously, due to the possibility that the intervention was not delivered as intended [49]. The ubiquitous publication of supplementary material can overcome space constraints in journals and can allow the publication of detailed intervention content, theories, behaviour change techniques and fidelity frameworks. Authors should increase the reporting clarity on the functional components of behaviour change interventions, which can only benefit behaviour change science, and public health more broadly [50].

The cost to carry out the interventions enabled comparison of included studies and to the broader literature. Mattli and colleagues conducted a review examining PA interventions for primary prevention [12]. The average costs of PA interventions were $US197 per/person (range $US25 to $US1,260 per/person); the majority of interventions in that study were not deemed cost-effective [12]. Workplace behaviour change interventions to increase PA averaged a cost of $US233 (range $US57 to $US682 per/person) [16]. It was inconclusive if worksite behaviour interventions were cost-effective strategies to increase PA [16]. The per person costs with from these reviews [12, 16] are similar to the $US313 (range $USS83 to $US1,298 per/person) found in this current review. These average costs per person are less expensive than the $US1,190 reported per intervention by Muller-Riemenschneider and colleagues [51]. While behaviour change interventions have made a significant contribution towards improving PA, these interventions generally come at a financial cost [52]. It is necessary that academics and policy makers are able to identify if the benefits of behaviour change interventions are greater than alternative options, and this is particularly telling for interventions aiming to increase PA. Alternative options may include policy, systems and/or environmental changes; future research may be undertaken to examine the cost-effectiveness of such methods versus in-person interventions.

One of the difficulties in determining if behaviour change interventions designed to improve PA are cost-effective is that evaluations of effectiveness depend on the willingness-to-pay for the intended change [53]. To our knowledge, there is no established willingness-to-pay threshold for PA change. Therefore, it is up to decision-makers to assess whether the ICERs for the PA outcomes represent good value for money, for example how much they are willing-to-pay for each additional minute of MVPA or MET-hour gained. Wu and colleagues [13] tried to address this uncertainty by providing a benchmark for costs per MET-hour gained. Three interventions in this current review showed value for money (improvements in PA versus intervention costs) with ICERs below the benchmark of US$0.5–US$1.00 per MET-hour gained [33, 34, 44]. Mattli and colleagues found four PA interventions that represented value for money [12]. In a systematic review of reviews, data drawn from eight reviews provided inconclusive evidence for cost-effectiveness of PA counselling interventions [54]. A large number of studies are carried out every year to investigate the effectiveness of behaviour change interventions to promote changes in PA [3, 6, 7]. Despite this, there is a relative scarcity of trial-based evaluations designed to examine the cost-effectiveness of behaviour change interventions. This scarcity is in keeping with the reported lack economic evidence for all interventions designed to promote changes in PA [55].

### Strengths and limitations

This is the first systematic review examining economic evaluations of behaviour change interventions designed to change PA in adults free-living in the community. To the best of our knowledge this was also the first review to attempt to summarise behaviour change intervention costs and outcomes, to conduct an in-depth appraisal of the risk of bias of the studies and to apply GRADE style rating to assess the certainty of the evidence.

This following limitations need to be considered. The included studies investigated different populations, settings, comparators, outcome measures and follow-up durations. The interventions were too heterogeneous to examine summary estimates using meta-analysis techniques [56]. To help compare studies, where possible, PA measures were standardised to MET-hours gained per person per day. The standardisation of PA outcomes included both device-measured and self-reported PA. The average MET-hours gained per person per day were higher in the studies using device-measured compared to self-report. Self-report measures of PA have been found to be higher and lower than device measured PA [57]. Many studies did not provide sufficient detail on PA outcomes and were unable to be included in the synthesis. To examine the cost-effectiveness, a benchmark chosen in previous studies was used [13, 14]. This benchmark may not be directly applicable to settings with different levels of insufficient PA or health care expenditure i.e. settings that might decide on different benchmarks for assessing cost-effectiveness of interventions to increase PA. Too address this uncertainty the authors conclusions on cost-effectiveness have also been reported on.

### Recommendations for future research

Future economic evaluations of behaviour change interventions designed to increase PA should describe the intervention in detail, including the type of theoretical framework used, behaviour change techniques, frequency and duration. In addition, detailed descriptions of the control intervention should be provided as this information can influence the interpretation of results.

Authors should provide a detailed breakdown of the components and associated costs involved in designing and implementing a behaviour change interventions designed to change PA, costs such as staff, training and equipment. Where possible, unit costs should also be provided for each item. The separate reporting of fixed and variable costs would facilitate costing implications when considering scaling-up of behaviour change interventions [58]. Authors should report disaggregated values for all included data, detailing the costs for all groups, the incremental costs and incremental outcomes. By providing disaggregated values instead of ICERs alone, the detailed information might improve the interpretation of findings and comparability to other studies. Authors should publish measures of uncertainty in the results by reporting uncertainty intervals and cost-effectiveness acceptability curves.

Agreeing on a common classification of PA outcomes for economic evaluation would improve the comparability of results across studies and reviews [54]. The value of conducting reviews into behaviour change interventions and PA interventions could be markedly improved if results were easily compared, and if possible, PA results were reported using agreed categorisations of outcome measures. In the absence of a common classification, researchers should consider measuring and reporting PA outcomes that allow for standardised conversion using the formula of Wu and colleagues [13]. These PA measures include outcomes such as steps/day or minutes/day of PA, and can be readily converted to MET-hours gained for broad comparisons [13].

## Conclusions

This review examined economic evaluations (*n* = 16, 14 of moderate certainty evidence) investigating the value for money of behaviour change interventions designed to increase PA. The included studies were heterogeneous in economic perspectives, follow-up time and populations, which is reflected in the variance in the overall outcomes of the economic analyses. In 75% of the studies the behaviour change interventions were reported as cost-effective methods for increasing PA. When examined against an applied benchmark for the cost per PA change, 38% of the interventions met the criteria for cost-effectiveness. The intervention costs summarised in this review varied and should be interpreted with a consideration for the number of sessions, duration and number of participants. The information in this review can be used for planning the implementation of future programmes or future models investigating the value for money of such programmes.

### Electronic supplementary material

Below is the link to the electronic supplementary material.

Supplementary Material 1

## Data Availability

The datasets used and/or analysed during the current study are available from the corresponding author on reasonable request.

## Abbreviations

CHEC: Consensus on Health Economic Criteria list
GRADE: Grading of Recommendations Assessment, Development, and Evaluation
ICER: Incremental Cost-effectiveness Ratio
MET: Metabolic Equivalent of Task
MVPA: Moderate to Vigorous Physical Activity
OECD: Organisation for Economic Co-operation and Development
PA: Physical Activity
PRISMA: Preferred Reporting Items for Systematic Reviews and Meta-Analyses
QALY: Quality Adjusted Life Year
