# Supplementary material for: Behaviour change interventions to improve physical activity in adults: a systematic review of economic evaluations
Source: Int J Behav Nutr Phys Act. 2024 Jul 9;21:73. doi: 10.1186/s12966-024-01614-6 (PMC11232201; doi:10.1186/s12966-024-01614-6)
Supplement: Supplementary file 1 — Supplementary Material 1 [file 12966_2024_1614_MOESM1_ESM.pdf]

Table of Contents: Additional files

Additional file 1. PRISMA Checklist 2

Additional file 2: Search strategy MEDLINE 5

Additional file 3. GRADE style rating for trial based economic evaluation of behaviour change interventions 7

Additional file 4. Completed GRADE style ratings for trial based economic evaluation of behaviour change interventions 10

Additional file 5. Records excluded at full-text screening and reasons for exclusion 58

Additional file 6. Risk of bias assessment of economic evaluations included in the review using the Consensus on Health Economic Criteria list (CHEC-list) 65

Additional file 1. PRISMA Checklist

Section and Topic	Item #	Checklist item	Location where item is reported
**TITLE**			
Title	1	Identify the report as a systematic review.	1
**ABSTRACT**			
Abstract	2	See the PRISMA 2020 for Abstracts checklist.	
**INTRODUCTION**			
Rationale	3	Describe the rationale for the review in the context of existing knowledge.	3-4
Objectives	4	Provide an explicit statement of the objective(s) or question(s) the review addresses.	4
**METHODS**			
Eligibility criteria	5	Specify the inclusion and exclusion criteria for the review and how studies were grouped for the syntheses.	5
Information sources	6	Specify all databases, registers, websites, organisations, reference lists and other sources searched or consulted to identify studies. Specify the date when each source was last searched or consulted.	4
Search strategy	7	Present the full search strategies for all databases, registers and websites, including any filters and limits used.	4
Selection process	8	Specify the methods used to decide whether a study met the inclusion criteria of the review, including how many reviewers screened each record and each report retrieved, whether they worked independently, and if applicable, details of automation tools used in the process.	4-5
Data collection process	9	Specify the methods used to collect data from reports, including how many reviewers collected data from each report, whether they worked independently, any processes for obtaining or confirming data from study investigators, and if applicable, details of automation tools used in the process.	6
Data items	10a	List and define all outcomes for which data were sought. Specify whether all results that were compatible with each outcome domain in each study were sought (e.g. for all measures, time points, analyses), and if not, the methods used to decide which results to collect.	5-6
	10b	List and define all other variables for which data were sought (e.g. participant and intervention characteristics, funding sources). Describe any assumptions made about any missing or unclear information.	5-6
Study risk of bias assessment	11	Specify the methods used to assess risk of bias in the included studies, including details of the tool(s) used, how many reviewers assessed each study and whether they worked independently, and if applicable, details of automation tools used in the process.	6-7
Effect measures	12	Specify for each outcome the effect measure(s) (e.g. risk ratio, mean difference) used in the synthesis or presentation of results.	7
Synthesis methods	13a	Describe the processes used to decide which studies were eligible for each synthesis (e.g. tabulating the study intervention characteristics and comparing against the planned groups for each synthesis (item #5)).	7
	13b	Describe any methods required to prepare the data for presentation or synthesis, such as handling of missing summary statistics, or data conversions.	7-9

Section and Topic	Item #	Checklist item	Location where item is reported
	13c	Describe any methods used to tabulate or visually display results of individual studies and syntheses.	7-9
	13d	Describe any methods used to synthesize results and provide a rationale for the choice(s). If meta-analysis was performed, describe the model(s), method(s) to identify the presence and extent of statistical heterogeneity, and software package(s) used.	7-9
	13e	Describe any methods used to explore possible causes of heterogeneity among study results (e.g. subgroup analysis, meta-regression).	7-9
	13f	Describe any sensitivity analyses conducted to assess robustness of the synthesized results.	7-9
Reporting bias assessment	14	Describe any methods used to assess risk of bias due to missing results in a synthesis (arising from reporting biases).	7-9
Certainty assessment	15	Describe any methods used to assess certainty (or confidence) in the body of evidence for an outcome.	7
**RESULTS**			
Study selection	16a	Describe the results of the search and selection process, from the number of records identified in the search to the number of studies included in the review, ideally using a flow diagram.	9
	16b	Cite studies that might appear to meet the inclusion criteria, but which were excluded, and explain why they were excluded.	9
Study characteristics	17	Cite each included study and present its characteristics.	9-11
Risk of bias in studies	18	Present assessments of risk of bias for each included study.	10
Results of individual studies	19	For all outcomes, present, for each study: (a) summary statistics for each group (where appropriate) and (b) an effect estimate and its precision (e.g. confidence/credible interval), ideally using structured tables or plots.	10-11
Results of syntheses	20a	For each synthesis, briefly summarise the characteristics and risk of bias among contributing studies.	10-11
	20b	Present results of all statistical syntheses conducted. If meta-analysis was done, present for each the summary estimate and its precision (e.g. confidence/credible interval) and measures of statistical heterogeneity. If comparing groups, describe the direction of the effect.	10-11
	20c	Present results of all investigations of possible causes of heterogeneity among study results.	10-12
	20d	Present results of all sensitivity analyses conducted to assess the robustness of the synthesized results.	NA
Reporting biases	21	Present assessments of risk of bias due to missing results (arising from reporting biases) for each synthesis assessed.	NA
Certainty of evidence	22	Present assessments of certainty (or confidence) in the body of evidence for each outcome assessed.	10
**DISCUSSION**			
Discussion	23a	Provide a general interpretation of the results in the context of other evidence.	14-16
	23b	Discuss any limitations of the evidence included in the review.	16-17
	23c	Discuss any limitations of the review processes used.	16-17

Section and Topic	Item #	Checklist item	Location where item is reported
	23d	Discuss implications of the results for practice, policy, and future research.	17-18
**OTHER INFORMATION**			
Registration and protocol	24a	Provide registration information for the review, including register name and registration number, or state that the review was not registered.	4
	24b	Indicate where the review protocol can be accessed, or state that a protocol was not prepared.	4
	24c	Describe and explain any amendments to information provided at registration or in the protocol.	4
Support	25	Describe sources of financial or non-financial support for the review, and the role of the funders or sponsors in the review.	27
Competing interests	26	Declare any competing interests of review authors.	27
Availability of data, code and other materials	27	Report which of the following are publicly available and where they can be found: template data collection forms; data extracted from included studies; data used for all analyses; analytic code; any other materials used in the review.	NA

Additional file 2: Search strategy MEDLINE

Ovid MEDLINE

Number	Terms	Retrieved
1	behav* change.mp. [mp=title, book title, abstract, original title, name of substance word, subject heading word, floating sub-heading word, keyword heading word, organism supplementary concept word, protocol supplementary concept word, rare disease supplementary concept word, unique identifier, synonyms, population supplementary concept word, anatomy supplementary concept word]	29161
2	motivation.mp. [mp=title, book title, abstract, original title, name of substance word, subject heading word, floating sub-heading word, keyword heading word, organism supplementary concept word, protocol supplementary concept word, rare disease supplementary concept word, unique identifier, synonyms, population supplementary concept word, anatomy supplementary concept word]	144965
3	lifestyle intervention.mp. [mp=title, book title, abstract, original title, name of substance word, subject heading word, floating sub-heading word, keyword heading word, organism supplementary concept word, protocol supplementary concept word, rare disease supplementary concept word, unique identifier, synonyms, population supplementary concept word, anatomy supplementary concept word]	6360
4	health coach*.mp. [mp=title, book title, abstract, original title, name of substance word, subject heading word, floating sub-heading word, keyword heading word, organism supplementary concept word, protocol supplementary concept word, rare disease supplementary concept word, unique identifier, synonyms, population supplementary concept word, anatomy supplementary concept word]	1439
5	1 or 2 or 3 or 4	177083
6	Cost-Benefit Analysis.mp. [mp=title, book title, abstract, original title, name of substance word, subject heading word, floating sub-heading word, keyword heading word, organism supplementary concept word, protocol supplementary concept word, rare disease supplementary concept word, unique identifier, synonyms, population supplementary concept word, anatomy supplementary concept word]	96465
7	economic evaluation.mp. [mp=title, book title, abstract, original title, name of substance word, subject heading word, floating sub-heading word, keyword heading word, organism supplementary concept word, protocol supplementary concept word, rare disease supplementary concept word, unique identifier, synonyms, population supplementary concept word, anatomy supplementary concept word]	13743
8	cost benefit analysis.mp. [mp=title, book title, abstract, original title, name of substance word, subject heading word, floating sub-heading word, keyword heading word, organism supplementary concept word, protocol supplementary concept word, rare disease supplementary concept word, unique identifier, synonyms, population supplementary concept word, anatomy supplementary concept word]	96465

9	exp Health Promotion/ or exp Health Care Costs/ or exp "Costs and Cost Analysis"/	351499
10	exp Health Care Costs/ or exp "Costs and Cost Analysis"/ or exp "Quality of Life"/ or cost utility analysis.mp.	535841
11	6 or 7 or 8 or 9 or 10	622519
12	physical activity.mp. or exp Physical Activity/	343683
13	exp Exercise/ or exercise.mp.	507739
14	exp Physical Activity/ or exp Lifestyle/ or exp Health Promotion/ or exp Health Behavior/ or sedentary lifestyle.mp.	670584
15	12 or 13 or 14	979571
16	5 and 11 and 15	9924
17	clinical trial.mp. or exp Clinical Trials/	807719
18	randomised controlled trial.mp.	33090
19	exp Clinical Trials/ or exp Randomized Controlled Trials/ or randomized controlled trial.mp.	807274
20	17 or 18 or 19	1292301
21	16 and 20	3071

Additional file 3. GRADE style rating for trial based economic evaluation of behaviour change interventions

Domain	Questions and sub-questions	Response	Notes
**A. Quality of trial-based economic evaluation reporting**	Is there a clear and comprehensive description of the methods?		
	1. Is the study population clearly described?	Yes = 1; No = 0	CHEC Q1
	2. Are competing alternatives clearly described?	Yes = 1; No = 0	CHEC Q2
	3. Is a well-defined research question posed in answerable form?	Yes = 1; No = 0	CHEC Q3
	4. Does the article indicate that there is no potential conflict of interest of	Yes = 1; No = 0	CHEC Q18
	**Rating for domain**	POOR/FAIR/ GOOD	0 or 1 = POOR; 2 or 3 = FAIR; 4 = GOOD
**B. Credibility of the clinical trial**	Has the trial generated appropriate and reliable inputs?		
	1. Were participants successfully randomised (i.e. participant characteristics well-matched at baseline)?	Yes = 1; No = 0	PEDro scale item 2 and 4
	2. Are the reported data complete (i.e. no concerns about missing data or participants being lost to follow-up)	Yes = 1; No = 0	PEDro scale item 2 and 4
	3. Were the interventions delivered as intended (i.e. measures of fidelity)?	Yes = 1; No = 0	
	4. Is the duration of the trial appropriate for the inclusion of all relevant costs and consequences?	Yes = 1; No = 0	CHEC Q5
	**Rating for domain**	POOR/FAIR/ GOOD	0 or 1 = POOR; 2 or 3 = FAIR; 4 = GOOD

Domain	Questions and sub-questions	Response	Notes
**C. Credibility of economic evaluation**	Has an appropriate approach been taken for the economic analysis and has the validity of this approach been explored?		
	1. Is the economic study design appropriate to the stated objective?	Yes = 1; No = 0	CHEC Q4
	2. Is the actual perspective chosen appropriate?	Yes = 1; No = 0	CHEC Q6
	3. Are all important and relevant costs for each alternative included	Yes = 1; No = 0	CHEC Q7
	4. Are all costs measured appropriately in physical units?	Yes = 1; No = 0	CHEC Q8
	5. Are costs valued appropriately?	Yes = 1; No = 0	CHEC Q9
	6. Is an incremental analysis of costs and outcomes of alternatives performed and clearly reported?	Yes = 1; No = 0	CHEC Q13
	7. Are all future costs and outcomes discounted appropriately?	Yes = 1; No = 0	CHEC Q14
	8. Are all important variables, whose values are uncertain, appropriately subjected to sensitivity analysis?	Yes = 1; No = 0	CHEC Q15
	**Rating for domain**	POOR/FAIR/ GOOD	0 to 3 = POOR; 4 to 7 = FAIR; 8 = GOOD
**D. Certainty of economic evaluation results**	Has the economic analysis generated meaningful outputs?		
	1. Do the conclusions follow from the data reported? Do the authors critically discuss their results including the impact of uncertainty and limitations of the evidence?	Yes = 1; No = 0	CHEC Q16
	2. Do the authors report any variability measures around their summary estimate of cost-effectiveness (e.g. confidence intervals, a cost-effectiveness acceptability curve)?	Yes = 1; No = 0	
	3. Does the study discuss the generalizability of the results to other settings and patient/client groups?	Yes = 1; No = 0	CHEC Q17
	4. Are ethical and distributional issues discussed appropriately?	Yes = 1; No = 0	CHEC Q19
	**Rating for domain**	POOR/FAIR/ GOOD	0 or 1 = POOR; 2 or 3 = FAIR; 4 = GOOD

Domain	Questions and sub-questions	Response	Notes
**E. Applicability**	1. Are the sociodemographic characteristics of the population similar to the population of interest?	Yes = 1; No = 0	Based on PICOS specific to
	2. Is the investigated intervention similar to the intervention(s) of interest, in particular, was the intervention aimed at fall prevention?	Yes = 1; No = 0	the question of interest
	3. Does the comparator in the model represent a world without the intervention?	Yes = 1; No = 0	Based on PICOS specific to
	4. Are the outcomes investigated appropriate for the review question (i.e. a fall-related outcome, or QOL)?	Yes = 1; No = 0	the question of interest
	**Rating for domain**	POOR/FAIR/ GOOD	0 or 1 = POOR; 2 or 3 = FAIR; 4 = GOOD

**Level of certainty**	**Definition**	**How it is derived**
HIGH	We are confident that the outputs from the economic evaluation are reliable for decision-making	All domains in Table 1 are rated Good
MODERATE	The outputs from the economic evaluation are likely to be reliable for decision making, but there is a possibility the outputs are not a reliable prediction of the cost-effectiveness of the intervention	All domains in Table 1 are rated Fair or higher
LOW	We have limited confidence that the outputs from the economic evaluation are reliable for decision-making	One domain in Table 1 is rated Poor but all other domains are rated Fair or Good
VERY LOW	We have very little confidence that the outputs from the economic evaluation are reliable for decision-making	More than one domain in Table 1 is rated Poor

Reasons to downgrade the overall level of certainty: If serious or very serious concerns exist for any of the above domains the evidence can be downgraded by one or two levels.
Reasons to upgrade the overall level of certainty: if the trial is judged to be at very low risk of bias in Domain B (e.g. well conducted and very large)

Additional file 4. Completed GRADE style ratings for trial based economic evaluation of behaviour change interventions

Barrett, 2019

Domain	Questions and sub-questions	Response	Notes
**A. Quality of trial-based economic evaluation reporting**	Is there a clear and comprehensive description of the methods?		
	1. Is the study population clearly described?	Yes	CHEC Q1
	2. Are competing alternatives clearly described?	Yes	CHEC Q2
	3. Is a well-defined research question posed in answerable form?	Yes	CHEC Q3
	4. Does the article indicate that there is no potential conflict of interest of	No	CHEC Q18
	**Rating for domain**	**FAIR**	0 or 1 = POOR; 2 or 3 = FAIR; 4 = GOOD
**B. Credibility of the clinical trial**	Has the trial generated appropriate and reliable inputs?		
	1. Were participants successfully randomised (i.e. participant characteristics well-matched at baseline)?	Yes	PEDro scale item 2 and 4
	2. Are the reported data complete (i.e. no concerns about missing data or participants being lost to follow-up)	Yes	PEDro scale item 2 and 4
	3. Were the interventions delivered as intended?	Yes	
	4. Is the duration of the trial appropriate for the inclusion of all relevant costs and consequences?	No	CHEC Q5
	**Rating for domain**	**FAIR**	0 or 1 = POOR; 2 or 3 = FAIR; 4 = GOOD

Domain	Questions and sub-questions	Response	Notes
**C. Credibility of economic evaluation**	Has an appropriate approach been taken for the economic analysis and has the validity of this approach been explored?		
	1. Is the economic study design appropriate to the stated objective?	Yes	CHEC Q4
	2. Is the actual perspective chosen appropriate?	No	CHEC Q6
	3. Are all important and relevant costs for each alternative included	Yes	CHEC Q7
	4. Are all costs measured appropriately in physical units?	Yes	CHEC Q8
	5. Are costs valued appropriately?	Yes	CHEC Q9
	6. Is an incremental analysis of costs and outcomes of alternatives performed and clearly reported?	Yes	CHEC Q13
	7. Are all future costs and outcomes discounted appropriately?	Yes	CHEC Q14
	8. Are all important variables, whose values are uncertain, appropriately subjected to sensitivity analysis?	Yes	CHEC Q15
	**Rating for domain**	**FAIR**	0 to 3 = POOR; 4 to 7 = FAIR; 8 = GOOD
**D. Certainty of economic evaluation results**	Has the economic analysis generated meaningful outputs?		
	1. Do the conclusions follow from the data reported? Do the authors critically discuss their results including the impact of uncertainty and limitations of the evidence?	No	CHEC Q16
	2. Do the authors report any variability measures around their summary estimate of cost-effectiveness (e.g. confidence intervals, a cost-effectiveness acceptability curve)?	Yes	
	3. Does the study discuss the generalizability of the results to other settings and patient/client groups?	Yes	CHEC Q17
	4. Are ethical and distributional issues discussed appropriately?	No	CHEC Q19
	**Rating for domain**	**FAIR**	0 or 1 = POOR; 2 or 3 = FAIR; 4 = GOOD

Domain	Questions and sub-questions	Response	Notes
**E. Applicability**	1. Are the sociodemographic characteristics of the population similar to the population of interest, in particular physically inactive individuals?	Yes	
	2. Is the investigated intervention similar to the intervention(s) of interest, in particular, was the intervention aimed at promoting physical activity behaviour change?	Yes	
	3. Are the outcomes investigated appropriate for the review question (i.e. a physical activity outcome, or QOL)?	Yes	
	**Rating for domain**	**GOOD**	0 or 1 = POOR; 2 = FAIR; 3 = GOOD

Level of certainty: MODERATE. The outputs from the economic evaluation are likely to be reliable for decision making, but there is a possibility the outputs are not a reliable prediction of the cost-effectiveness of the intervention

Barrett, 2022

Domain	Questions and sub-questions	Response	Notes
**A. Quality of trial-based economic evaluation reporting**	Is there a clear and comprehensive description of the methods?		
	1. Is the study population clearly described?	Yes	CHEC Q1
	2. Are competing alternatives clearly described?	Yes	CHEC Q2
	3. Is a well-defined research question posed in answerable form?	Yes	CHEC Q3
	4. Does the article indicate that there is no potential conflict of interest of	Yes	CHEC Q18
	**Rating for domain**	**GOOD**	0 or 1 = POOR; 2 or 3 = FAIR; 4 = GOOD
**B. Credibility of the clinical trial**	Has the trial generated appropriate and reliable inputs?		
	1. Were participants successfully randomised (i.e. participant characteristics well-matched at baseline)?	Yes	PEDro scale item 2 and 4
	2. Are the reported data complete (i.e. no concerns about missing data or participants being lost to follow-up)	Yes	PEDro scale item 2 and 4
	3. Were the interventions delivered as intended?	Yes	
	4. Is the duration of the trial appropriate for the inclusion of all relevant costs and consequences?	YES	CHEC Q5
	**Rating for domain**	**GOOD**	0 or 1 = POOR; 2 or 3 = FAIR; 4 = GOOD

Domain	Questions and sub-questions	Response	Notes
**C. Credibility of economic evaluation**	Has an appropriate approach been taken for the economic analysis and has the validity of this approach been explored?		
	1. Is the economic study design appropriate to the stated objective?	Yes	CHEC Q4
	2. Is the actual perspective chosen appropriate?	No	CHEC Q6
	3. Are all important and relevant costs for each alternative included	Yes	CHEC Q7
	4. Are all costs measured appropriately in physical units?	Yes	CHEC Q8
	5. Are costs valued appropriately?	Yes	CHEC Q9
	6. Is an incremental analysis of costs and outcomes of alternatives performed and clearly reported?	Yes	CHEC Q13
	7. Are all future costs and outcomes discounted appropriately?	No	CHEC Q14
	8. Are all important variables, whose values are uncertain, appropriately subjected to sensitivity analysis?	Yes	CHEC Q15
	**Rating for domain**	**FAIR**	0 to 3 = POOR; 4 to 7 = FAIR; 8 = GOOD
**D. Certainty of economic evaluation results**	Has the economic analysis generated meaningful outputs?		
	1. Do the conclusions follow from the data reported? Do the authors critically discuss their results including the impact of uncertainty and limitations of the evidence?	Yes	CHEC Q16
	2. Do the authors report any variability measures around their summary estimate of cost-effectiveness (e.g. confidence intervals, a cost-effectiveness acceptability curve)?	Yes	
	3. Does the study discuss the generalizability of the results to other settings and patient/client groups?	Yes	CHEC Q17
	4. Are ethical and distributional issues discussed appropriately?	Yes	CHEC Q19
	**Rating for domain**	**GOOD**	0 or 1 = POOR; 2 or 3 = FAIR; 4 = GOOD

Domain	Questions and sub-questions	Response	Notes
**E. Applicability** Domain	1. Are the sociodemographic characteristics of the population similar to the population of interest, in particular physically inactive individuals?	Yes	
	2. Is the investigated intervention similar to the intervention(s) of interest, in particular, was the intervention aimed at promoting physical activity behaviour change?	Yes	
	3. Are the outcomes investigated appropriate for the review question (i.e. a physical activity outcome, or QOL)?	Yes	
	**Rating for domain**	**GOOD**	0 or 1 = POOR; 2 = FAIR; 3 = GOOD
	Questions and sub-questions	Response	Notes

Level of certainty: MODERATE. The outputs from the economic evaluation are likely to be reliable for decision making, but there is a possibility the outputs are not a reliable prediction of the cost-effectiveness of the intervention

Brodin, 2015

Domain	Questions and sub-questions	Response	Notes
**A. Quality of trial-based economic evaluation reporting**	Is there a clear and comprehensive description of the methods?		
	1. Is the study population clearly described?	Yes	CHEC Q1
	2. Are competing alternatives clearly described?	No	CHEC Q2
	3. Is a well-defined research question posed in answerable form?	Yes	CHEC Q3
	4. Does the article indicate that there is no potential conflict of interest of	Yes	CHEC Q18
	**Rating for domain**	**FAIR**	0 or 1 = POOR; 2 or 3 = FAIR; 4 = GOOD
**B. Credibility of the clinical trial**	Has the trial generated appropriate and reliable inputs?		
	1. Were participants successfully randomised (i.e. participant characteristics well-matched at baseline)?	Yes	PEDro scale item 2 and 4
	2. Are the reported data complete (i.e. no concerns about missing data or participants being lost to follow-up)	Yes	PEDro scale item 2 and 4
	3. Were the interventions delivered as intended?	Yes	
	4. Is the duration of the trial appropriate for the inclusion of all relevant costs and consequences?	No	CHEC Q5
	**Rating for domain**	**FAIR**	0 or 1 = POOR; 2 or 3 = FAIR; 4 = GOOD

Domain	Questions and sub-questions	Response	Notes
**C. Credibility of economic evaluation**	Has an appropriate approach been taken for the economic analysis and has the validity of this approach been explored?		
	1. Is the economic study design appropriate to the stated objective?	Yes	CHEC Q4
	2. Is the actual perspective chosen appropriate?	No	CHEC Q6
	3. Are all important and relevant costs for each alternative included	Yes	CHEC Q7
	4. Are all costs measured appropriately in physical units?	Yes	CHEC Q8
	5. Are costs valued appropriately?	Yes	CHEC Q9
	6. Is an incremental analysis of costs and outcomes of alternatives performed and clearly reported?	Yes	CHEC Q13
	7. Are all future costs and outcomes discounted appropriately?	No	CHEC Q14
	8. Are all important variables, whose values are uncertain, appropriately subjected to sensitivity analysis?	Yes	CHEC Q15
	**Rating for domain**	**FAIR**	0 to 3 = POOR; 4 to 7 = FAIR; 8 = GOOD
**D. Certainty of economic evaluation results**	Has the economic analysis generated meaningful outputs?		
	1. Do the conclusions follow from the data reported? Do the authors critically discuss their results including the impact of uncertainty and limitations of the evidence?	Yes	CHEC Q16
	2. Do the authors report any variability measures around their summary estimate of cost-effectiveness (e.g. confidence intervals, a cost-effectiveness acceptability curve)?	No	
	3. Does the study discuss the generalizability of the results to other settings and patient/client groups?	Yes	CHEC Q17
	4. Are ethical and distributional issues discussed appropriately?	No	CHEC Q19
	**Rating for domain**	**FAIR**	0 or 1 = POOR; 2 or 3 = FAIR; 4 = GOOD

Domain	Questions and sub-questions	Response	Notes
**E. Applicability** Domain	1. Are the sociodemographic characteristics of the population similar to the population of interest, in particular physically inactive individuals?	No	Not specified that they were insufficiently active at baseline
	2. Is the investigated intervention similar to the intervention(s) of interest, in particular, was the intervention aimed at promoting physical activity behaviour change?	Yes	
	3. Are the outcomes investigated appropriate for the review question (i.e. a physical activity outcome, or QOL)?	Yes	
	**Rating for domain**	**FAIR**	0 or 1 = POOR; 2 = FAIR; 3 = GOOD
	Questions and sub-questions	Response	Notes

Level of certainty: MODERATE. The outputs from the economic evaluation are likely to be reliable for decision making, but there is a possibility the outputs are not a reliable prediction of the cost-effectiveness of the intervention

Broekhuizen, 2018

Domain	Questions and sub-questions	Response	Notes
**A. Quality of trial-based economic evaluation reporting**	Is there a clear and comprehensive description of the methods?		
	1. Is the study population clearly described?	Yes	CHEC Q1
	2. Are competing alternatives clearly described?	No	CHEC Q2
	3. Is a well-defined research question posed in answerable form?	Yes	CHEC Q3
	4. Does the article indicate that there is no potential conflict of interest of	Yes	CHEC Q18
	**Rating for domain**	**FAIR**	0 or 1 = POOR; 2 or 3 = FAIR; 4 = GOOD
**B. Credibility of the clinical trial**	Has the trial generated appropriate and reliable inputs?		
	1. Were participants successfully randomised (i.e. participant characteristics well-matched at baseline)?	Yes	PEDro scale item 2 and 4
	2. Are the reported data complete (i.e. no concerns about missing data or participants being lost to follow-up)	No	PEDro scale item 2 and 4
	3. Were the interventions delivered as intended?	Yes	
	4. Is the duration of the trial appropriate for the inclusion of all relevant costs and consequences?	Yes	CHEC Q5
	**Rating for domain**	**FAIR**	0 or 1 = POOR; 2 or 3 = FAIR; 4 = GOOD

Domain	Questions and sub-questions	Response	Notes
**C. Credibility of economic evaluation**	Has an appropriate approach been taken for the economic analysis and has the validity of this approach been explored?		
	1. Is the economic study design appropriate to the stated objective?	Yes	CHEC Q4
	2. Is the actual perspective chosen appropriate?	Yes	CHEC Q6
	3. Are all important and relevant costs for each alternative included	Yes	CHEC Q7
	4. Are all costs measured appropriately in physical units?	Yes	CHEC Q8
	5. Are costs valued appropriately?	Yes	CHEC Q9
	6. Is an incremental analysis of costs and outcomes of alternatives performed and clearly reported?	Yes	CHEC Q13
	7. Are all future costs and outcomes discounted appropriately?	No	CHEC Q14
	8. Are all important variables, whose values are uncertain, appropriately subjected to sensitivity analysis?	Yes	CHEC Q15
	**Rating for domain**	**FAIR**	0 to 3 = POOR; 4 to 7 = FAIR; 8 = GOOD
**D. Certainty of economic evaluation results**	Has the economic analysis generated meaningful outputs?		
	1. Do the conclusions follow from the data reported? Do the authors critically discuss their results including the impact of uncertainty and limitations of the evidence?	Yes	CHEC Q16
	2. Do the authors report any variability measures around their summary estimate of cost-effectiveness (e.g. confidence intervals, a cost-effectiveness acceptability curve)?	Yes	
	3. Does the study discuss the generalizability of the results to other settings and patient/client groups?	No	CHEC Q17
	4. Are ethical and distributional issues discussed appropriately?	No	CHEC Q19
	**Rating for domain**	**FAIR**	0 or 1 = POOR; 2 or 3 = FAIR; 4 = GOOD

Domain	Questions and sub-questions	Response	Notes
**E. Applicability**<br>Domain	1. Are the sociodemographic characteristics of the population similar to the population of interest, in particular physically inactive individuals?	No	Not specified that they were insufficiently active at baseline
	2. Is the investigated intervention similar to the intervention(s) of interest, in particular, was the intervention aimed at promoting physical activity behaviour change?	Yes	
	3. Are the outcomes investigated appropriate for the review question (i.e. a physical activity outcome, or QOL)?	Yes	
	**Rating for domain**	**FAIR**	0 or 1 = POOR; 2 = FAIR;<br>3 = GOOD
	Questions and sub-questions	Response	Notes

Level of certainty: MODERATE. The outputs from the economic evaluation are likely to be reliable for decision making, but there is a possibility the outputs are not a reliable prediction of the cost-effectiveness of the intervention

Buder, 2018

Domain	Questions and sub-questions	Response	Notes
**A. Quality of trial-based economic evaluation reporting**	Is there a clear and comprehensive description of the methods?		
	1. Is the study population clearly described?	Yes	CHEC Q1
	2. Are competing alternatives clearly described?	Yes	CHEC Q2
	3. Is a well-defined research question posed in answerable form?	Yes	CHEC Q3
	4. Does the article indicate that there is no potential conflict of interest of	No	CHEC Q18
	**Rating for domain**	**FAIR**	0 or 1 = POOR; 2 or 3 = FAIR; 4 = GOOD
**B. Credibility of the clinical trial**	Has the trial generated appropriate and reliable inputs?		
	1. Were participants successfully randomised (i.e. participant characteristics well-matched at baseline)?	Yes	PEDro scale item 2 and 4
	2. Are the reported data complete (i.e. no concerns about missing data or participants being lost to follow-up)	Yes	PEDro scale item 2 and 4
	3. Were the interventions delivered as intended?	Yes	
	4. Is the duration of the trial appropriate for the inclusion of all relevant costs and consequences?	Yes	CHEC Q5
	**Rating for domain**	**GOOD**	0 or 1 = POOR; 2 or 3 = FAIR; 4 = GOOD

Domain	Questions and sub-questions	Response	Notes
**C. Credibility of economic evaluation**	Has an appropriate approach been taken for the economic analysis and has the validity of this approach been explored?		
	1. Is the economic study design appropriate to the stated objective?	No	CHEC Q4
	2. Is the actual perspective chosen appropriate?	No	CHEC Q6
	3. Are all important and relevant costs for each alternative included	Yes	CHEC Q7
	4. Are all costs measured appropriately in physical units?	Yes	CHEC Q8
	5. Are costs valued appropriately?	No	CHEC Q9
	6. Is an incremental analysis of costs and outcomes of alternatives performed and clearly reported?	No	CHEC Q13
	7. Are all future costs and outcomes discounted appropriately?	No	CHEC Q14
	8. Are all important variables, whose values are uncertain, appropriately subjected to sensitivity analysis?	Yes	CHEC Q15
	**Rating for domain**	**POOR**	0 to 3 = POOR; 4 to 7 = FAIR; 8 = GOOD
**D. Certainty of economic evaluation results**	Has the economic analysis generated meaningful outputs?		
	1. Do the conclusions follow from the data reported? Do the authors critically discuss their results including the impact of uncertainty and limitations of the evidence?	Yes	CHEC Q16
	2. Do the authors report any variability measures around their summary estimate of cost-effectiveness (e.g. confidence intervals, a cost-effectiveness acceptability curve)?	No	
	3. Does the study discuss the generalizability of the results to other settings and patient/client groups?	Yes	CHEC Q17
	4. Are ethical and distributional issues discussed appropriately?	No	CHEC Q19
	**Rating for domain**	**FAIR**	0 or 1 = POOR; 2 or 3 = FAIR; 4 = GOOD

Domain	Questions and sub-questions	Response	Notes
**E. Applicability** Domain	1. Are the sociodemographic characteristics of the population similar to the population of interest, in particular physically inactive individuals?	No	Not specified that they were insufficiently active at baseline
	2. Is the investigated intervention similar to the intervention(s) of interest, in particular, was the intervention aimed at promoting physical activity behaviour change?	Yes	
	3. Are the outcomes investigated appropriate for the review question (i.e. a physical activity outcome, or QOL)?	Yes	
	**Rating for domain**	**FAIR**	0 or 1 = POOR; 2 = FAIR; 3 = GOOD
	Questions and sub-questions	Response	Notes

Level of certainty: LOW. We have limited confidence that the outputs from the economic evaluation are reliable for decision-making

Crist, 2022

Domain	Questions and sub-questions	Response	Notes
**A. Quality of trial-based economic evaluation reporting**	Is there a clear and comprehensive description of the methods?		
	1. Is the study population clearly described?	Yes	CHEC Q1
	2. Are competing alternatives clearly described?	No	CHEC Q2
	3. Is a well-defined research question posed in answerable form?	Yes	CHEC Q3
	4. Does the article indicate that there is no potential conflict of interest of	No	CHEC Q18
	**Rating for domain**	**FAIR**	0 or 1 = POOR; 2 or 3 = FAIR; 4 = GOOD
**B. Credibility of the clinical trial**	Has the trial generated appropriate and reliable inputs?		
	1. Were participants successfully randomised (i.e. participant characteristics well-matched at baseline)?	Yes	PEDro scale item 2 and 4
	2. Are the reported data complete (i.e. no concerns about missing data or participants being lost to follow-up)	Yes	PEDro scale item 2 and 4
	3. Were the interventions delivered as intended?	Yes	
	4. Is the duration of the trial appropriate for the inclusion of all relevant costs and consequences?	Yes	CHEC Q5
	**Rating for domain**	**GOOD**	0 or 1 = POOR; 2 or 3 = FAIR; 4 = GOOD

Domain	Questions and sub-questions	Response	Notes
**C. Credibility of economic evaluation**	Has an appropriate approach been taken for the economic analysis and has the validity of this approach been explored?		
	1. Is the economic study design appropriate to the stated objective?	Yes	CHEC Q4
	2. Is the actual perspective chosen appropriate?	No	CHEC Q6
	3. Are all important and relevant costs for each alternative included	Yes	CHEC Q7
	4. Are all costs measured appropriately in physical units?	Yes	CHEC Q8
	5. Are costs valued appropriately?	No	CHEC Q9
	6. Is an incremental analysis of costs and outcomes of alternatives performed and clearly reported?	Yes	CHEC Q13
	7. Are all future costs and outcomes discounted appropriately?	No	CHEC Q14
	8. Are all important variables, whose values are uncertain, appropriately subjected to sensitivity analysis?	No	CHEC Q15
	**Rating for domain**	**FAIR**	0 to 3 = POOR; 4 to 7 = FAIR; 8 = GOOD
**D. Certainty of economic evaluation results**	Has the economic analysis generated meaningful outputs?		
	1. Do the conclusions follow from the data reported? Do the authors critically discuss their results including the impact of uncertainty and limitations of the evidence?	Yes	CHEC Q16
	2. Do the authors report any variability measures around their summary estimate of cost-effectiveness (e.g. confidence intervals, a cost-effectiveness acceptability curve)?	No	
	3. Does the study discuss the generalizability of the results to other settings and patient/client groups?	Yes	CHEC Q17
	4. Are ethical and distributional issues discussed appropriately?	No	CHEC Q19
	**Rating for domain**	**FAIR**	0 or 1 = POOR; 2 or 3 = FAIR; 4 = GOOD

Domain	Questions and sub-questions	Response	Notes
**E. Applicability** Domain	1. Are the sociodemographic characteristics of the population similar to the population of interest, in particular physically inactive individuals?	No	Not specified that they were insufficiently active at baseline
	2. Is the investigated intervention similar to the intervention(s) of interest, in particular, was the intervention aimed at promoting physical activity behaviour change?	Yes	
	3. Are the outcomes investigated appropriate for the review question (i.e. a physical activity outcome, or QOL)?	Yes	
	**Rating for domain**	**FAIR**	0 or 1 = POOR; 2 = FAIR; 3 = GOOD
	Questions and sub-questions	Response	Notes

Level of certainty: MODERATE. The outputs from the economic evaluation are likely to be reliable for decision making, but there is a possibility the outputs are not a reliable prediction of the cost-effectiveness of the intervention.

Goyder, 2014

Domain	Questions and sub-questions	Response	Notes
**A. Quality of trial-based economic evaluation reporting**	Is there a clear and comprehensive description of the methods?		
	1. Is the study population clearly described?	Yes	CHEC Q1
	2. Are competing alternatives clearly described?	Yes	CHEC Q2
	3. Is a well-defined research question posed in answerable form?	Yes	CHEC Q3
	4. Does the article indicate that there is no potential conflict of interest of	Yes	CHEC Q18
	**Rating for domain**	**GOOD**	0 or 1 = POOR; 2 or 3 = FAIR; 4 = GOOD
**B. Credibility of the clinical trial**	Has the trial generated appropriate and reliable inputs?		
	1. Were participants successfully randomised (i.e. participant characteristics well-matched at baseline)?	Yes	
	2. Are the reported data complete (i.e. no concerns about missing data or participants being lost to follow-up)	Yes	
	3. Were the interventions delivered as intended?	Yes	
	4. Is the duration of the trial appropriate for the inclusion of all relevant costs and consequences?	Yes	CHEC Q5
	**Rating for domain**	**GOOD**	0 or 1 = POOR; 2 or 3 = FAIR; 4 = GOOD

Domain	Questions and sub-questions	Response	Notes
**C. Credibility of economic evaluation**	Has an appropriate approach been taken for the economic analysis and has the validity of this approach been explored?		
	1. Is the economic study design appropriate to the stated objective?	Yes	CHEC Q4
	2. Is the actual perspective chosen appropriate?	Yes	CHEC Q6
	3. Are all important and relevant costs for each alternative included	Yes	CHEC Q7
	4. Are all costs measured appropriately in physical units?	Yes	CHEC Q8
	5. Are costs valued appropriately?	Yes	CHEC Q9
	6. Is an incremental analysis of costs and outcomes of alternatives performed and clearly reported?	Yes	CHEC Q13
	7. Are all future costs and outcomes discounted appropriately?	No	CHEC Q14
	8. Are all important variables, whose values are uncertain, appropriately subjected to sensitivity analysis?	No	CHEC Q15
	**Rating for domain**	**GOOD**	0 to 3 = POOR; 4 to 7 = FAIR; 8 = GOOD
**D. Certainty of economic evaluation results**	Has the economic analysis generated meaningful outputs?		
	1. Do the conclusions follow from the data reported? Do the authors critically discuss their results including the impact of uncertainty and limitations of the evidence?	Yes	CHEC Q16
	2. Do the authors report any variability measures around their summary estimate of cost-effectiveness (e.g. confidence intervals, a cost-effectiveness acceptability curve)?	Yes	
	3. Does the study discuss the generalizability of the results to other settings and patient/client groups?	Yes	CHEC Q17
	4. Are ethical and distributional issues discussed appropriately?	Yes	CHEC Q19
	**Rating for domain**	**GOOD**	0 or 1 = POOR; 2 or 3 = FAIR; 4 = GOOD

Domain	Questions and sub-questions	Response	Notes
**E. Applicability** Domain	1. Are the sociodemographic characteristics of the population similar to the population of interest, in particular physically inactive individuals?	Yes	
	2. Is the investigated intervention similar to the intervention(s) of interest, in particular, was the intervention aimed at promoting physical activity behaviour change?	Yes	
	3. Are the outcomes investigated appropriate for the review question (i.e. a physical activity outcome, or QOL)?	Yes	
	**Rating for domain**	**Good**	0 or 1 = POOR; 2 = FAIR; 3 = GOOD
	Questions and sub-questions	Response	Notes

Level of certainty: HIGH. We are confident that the outputs from the economic evaluation are reliable for decision-making

Ismail, 2020

Domain	Questions and sub-questions	Response	Notes
**A. Quality of trial-based economic evaluation reporting**	Is there a clear and comprehensive description of the methods?		
	1. Is the study population clearly described?	Yes	CHEC Q1
	2. Are competing alternatives clearly described?	Yes	CHEC Q2
	3. Is a well-defined research question posed in answerable form?	Yes	CHEC Q3
	4. Does the article indicate that there is no potential conflict of interest of	No	CHEC Q18
	**Rating for domain**	**FAIR**	0 or 1 = POOR; 2 or 3 = FAIR; 4 = GOOD
**B. Credibility of the clinical trial**	Has the trial generated appropriate and reliable inputs?		
	1. Were participants successfully randomised (i.e. participant characteristics well-matched at baseline)?	Yes	
	2. Are the reported data complete (i.e. no concerns about missing data or participants being lost to follow-up)	Yes	
	3. Were the interventions delivered as intended?	Yes	
	4. Is the duration of the trial appropriate for the inclusion of all relevant costs and consequences?	Yes	CHEC Q5
	**Rating for domain**	**GOOD**	0 or 1 = POOR; 2 or 3 = FAIR; 4 = GOOD

Domain	Questions and sub-questions	Response	Notes
**C. Credibility of economic evaluation**	Has an appropriate approach been taken for the economic analysis and has the validity of this approach been explored?		
	1. Is the economic study design appropriate to the stated objective?	Yes	CHEC Q4
	2. Is the actual perspective chosen appropriate?	No	CHEC Q6
	3. Are all important and relevant costs for each alternative included	Yes	CHEC Q7
	4. Are all costs measured appropriately in physical units?	No	CHEC Q8
	5. Are costs valued appropriately?	Yes	CHEC Q9
	6. Is an incremental analysis of costs and outcomes of alternatives performed and clearly reported?	Yes	CHEC Q13
	7. Are all future costs and outcomes discounted appropriately?	No	CHEC Q14
	8. Are all important variables, whose values are uncertain, appropriately subjected to sensitivity analysis?	Yes	CHEC Q15
	**Rating for domain**	**FAIR**	0 to 3 = POOR; 4 to 7 = FAIR; 8 = GOOD
**D. Certainty of economic evaluation results**	Has the economic analysis generated meaningful outputs?		
	1. Do the conclusions follow from the data reported? Do the authors critically discuss their results including the impact of uncertainty and limitations of the evidence?	Yes	CHEC Q16
	2. Do the authors report any variability measures around their summary estimate of cost-effectiveness (e.g. confidence intervals, a cost-effectiveness acceptability curve)?	No	
	3. Does the study discuss the generalizability of the results to other settings and patient/client groups?	Yes	CHEC Q17
	4. Are ethical and distributional issues discussed appropriately?	Yes	CHEC Q19
	**Rating for domain**	**FAIR**	0 or 1 = POOR; 2 or 3 = FAIR; 4 = GOOD

Domain	Questions and sub-questions	Response	Notes
**E. Applicability** Domain	1. Are the sociodemographic characteristics of the population similar to the population of interest, in particular physically inactive individuals?	No	
	2. Is the investigated intervention similar to the intervention(s) of interest, in particular, was the intervention aimed at promoting physical activity behaviour change?	Yes	
	3. Are the outcomes investigated appropriate for the review question (i.e. a physical activity outcome, or QOL)?	Yes	
	**Rating for domain**	**Fair**	0 or 1 = POOR; 2 = FAIR; 3 = GOOD
	Questions and sub-questions	Response	Notes

Level of certainty: MODERATE. The outputs from the economic evaluation are likely to be reliable for decision making, but there is a possibility the outputs are not a reliable prediction of the cost-effectiveness of the intervention.

Jacobs, 2010

Domain	Questions and sub-questions	Response	Notes
**A. Quality of trial-based economic evaluation reporting**	Is there a clear and comprehensive description of the methods?		
	1. Is the study population clearly described?	Yes	CHEC Q1
	2. Are competing alternatives clearly described?	Yes	CHEC Q2
	3. Is a well-defined research question posed in answerable form?	Yes	CHEC Q3
	4. Does the article indicate that there is no potential conflict of interest of	No	CHEC Q18
	**Rating for domain**	**FAIR**	0 or 1 = POOR; 2 or 3 = FAIR; 4 = GOOD
**B. Credibility of the clinical trial**	Has the trial generated appropriate and reliable inputs?		
	1. Were participants successfully randomised (i.e. participant characteristics well-matched at baseline)?	Yes	
	2. Are the reported data complete (i.e. no concerns about missing data or participants being lost to follow-up)	Yes	
	3. Were the interventions delivered as intended?	Yes	
	4. Is the duration of the trial appropriate for the inclusion of all relevant costs and consequences?	Yes	CHEC Q5
	**Rating for domain**	**GOOD**	0 or 1 = POOR; 2 or 3 = FAIR; 4 = GOOD

Domain	Questions and sub-questions	Response	Notes
**C. Credibility of economic evaluation**	Has an appropriate approach been taken for the economic analysis and has the validity of this approach been explored?		
	1. Is the economic study design appropriate to the stated objective?	Yes	CHEC Q4
	2. Is the actual perspective chosen appropriate?	No	CHEC Q6
	3. Are all important and relevant costs for each alternative included	Yes	CHEC Q7
	4. Are all costs measured appropriately in physical units?	Yes	CHEC Q8
	5. Are costs valued appropriately?	Yes	CHEC Q9
	6. Is an incremental analysis of costs and outcomes of alternatives performed and clearly reported?	Yes	CHEC Q13
	7. Are all future costs and outcomes discounted appropriately?	No	CHEC Q14
	8. Are all important variables, whose values are uncertain, appropriately subjected to sensitivity analysis?	Yes	CHEC Q15
	**Rating for domain**	**FAIR**	0 to 3 = POOR; 4 to 7 = FAIR; 8 = GOOD
**D. Certainty of economic evaluation results**	Has the economic analysis generated meaningful outputs?		
	1. Do the conclusions follow from the data reported? Do the authors critically discuss their results including the impact of uncertainty and limitations of the evidence?	Yes	CHEC Q16
	2. Do the authors report any variability measures around their summary estimate of cost-effectiveness (e.g. confidence intervals, a cost-effectiveness acceptability curve)?	Yes	
	3. Does the study discuss the generalizability of the results to other settings and patient/client groups?	Yes	CHEC Q17
	4. Are ethical and distributional issues discussed appropriately?	Yes	CHEC Q19
	**Rating for domain**	**GOOD**	0 or 1 = POOR; 2 or 3 = FAIR; 4 = GOOD

Domain	Questions and sub-questions	Response	Notes
**E. Applicability** Domain	1. Are the sociodemographic characteristics of the population similar to the population of interest, in particular physically inactive individuals?	No	
	2. Is the investigated intervention similar to the intervention(s) of interest, in particular, was the intervention aimed at promoting physical activity behaviour change?	Yes	
	3. Are the outcomes investigated appropriate for the review question (i.e. a physical activity outcome, or QOL)?	Yes	
	**Rating for domain**	**Fair**	0 or 1 = POOR; 2 = FAIR; 3 = GOOD
	Questions and sub-questions	Response	Notes

Level of certainty: MODERATE. The outputs from the economic evaluation are likely to be reliable for decision making, but there is a possibility the outputs are not a reliable prediction of the cost-effectiveness of the intervention

Khunti, 2021

Domain	Questions and sub-questions	Response	Notes
**A. Quality of trial-based economic evaluation reporting**	Is there a clear and comprehensive description of the methods?		
	1. Is the study population clearly described?	Yes	CHEC Q1
	2. Are competing alternatives clearly described?	Yes	CHEC Q2
	3. Is a well-defined research question posed in answerable form?	Yes	CHEC Q3
	4. Does the article indicate that there is no potential conflict of interest of	No	CHEC Q18
	**Rating for domain**	**FAIR**	0 or 1 = POOR; 2 or 3 = FAIR; 4 = GOOD
**B. Credibility of the clinical trial**	Has the trial generated appropriate and reliable inputs?		
	1. Were participants successfully randomised (i.e. participant characteristics well-matched at baseline)?	Yes	
	2. Are the reported data complete (i.e. no concerns about missing data or participants being lost to follow-up)	Yes	
	3. Were the interventions delivered as intended?	Yes	
	4. Is the duration of the trial appropriate for the inclusion of all relevant costs and consequences?	Yes	CHEC Q5
	**Rating for domain**	**GOOD**	0 or 1 = POOR; 2 or 3 = FAIR; 4 = GOOD

Domain	Questions and sub-questions	Response	Notes
**C. Credibility of economic evaluation**	Has an appropriate approach been taken for the economic analysis and has the validity of this approach been explored?		
	1. Is the economic study design appropriate to the stated objective?	Yes	CHEC Q4
	2. Is the actual perspective chosen appropriate?	Yes	CHEC Q6
	3. Are all important and relevant costs for each alternative included	Yes	CHEC Q7
	4. Are all costs measured appropriately in physical units?	Yes	CHEC Q8
	5. Are costs valued appropriately?	Yes	CHEC Q9
	6. Is an incremental analysis of costs and outcomes of alternatives performed and clearly reported?	Yes	CHEC Q13
	7. Are all future costs and outcomes discounted appropriately?	Yes	CHEC Q14
	8. Are all important variables, whose values are uncertain, appropriately subjected to sensitivity analysis?	Yes	CHEC Q15
	**Rating for domain**	**GOOD**	0 to 3 = POOR; 4 to 7 = FAIR; 8 = GOOD
**D. Certainty of economic evaluation results**	Has the economic analysis generated meaningful outputs?		
	1. Do the conclusions follow from the data reported? Do the authors critically discuss their results including the impact of uncertainty and limitations of the evidence?	Yes	CHEC Q16
	2. Do the authors report any variability measures around their summary estimate of cost-effectiveness (e.g. confidence intervals, a cost-effectiveness acceptability curve)?	Yes	
	3. Does the study discuss the generalizability of the results to other settings and patient/client groups?	Yes	CHEC Q17
	4. Are ethical and distributional issues discussed appropriately?	Yes	CHEC Q19
	**Rating for domain**	**GOOD**	0 or 1 = POOR; 2 or 3 = FAIR; 4 = GOOD

Domain	Questions and sub-questions	Response	Notes
**E. Applicability** Domain	1. Are the sociodemographic characteristics of the population similar to the population of interest, in particular physically inactive individuals?	No	
	2. Is the investigated intervention similar to the intervention(s) of interest, in particular, was the intervention aimed at promoting physical activity behaviour change?	Yes	
	3. Are the outcomes investigated appropriate for the review question (i.e. a physical activity outcome, or QOL)?	Yes	
	**Rating for domain**	**Fair**	0 or 1 = POOR; 2 = FAIR; 3 = GOOD
	Questions and sub-questions	Response	Notes

Level of certainty: MODERATE. The outputs from the economic evaluation are likely to be reliable for decision making, but there is a possibility the outputs are not a reliable prediction of the cost-effectiveness of the intervention

Sangster, 2015

Domain	Questions and sub-questions	Response	Notes
**A. Quality of trial-based economic evaluation reporting**	Is there a clear and comprehensive description of the methods?		
	1. Is the study population clearly described?	Yes	CHEC Q1
	2. Are competing alternatives clearly described?	Yes	CHEC Q2
	3. Is a well-defined research question posed in answerable form?	Yes	CHEC Q3
	4. Does the article indicate that there is no potential conflict of interest of	Yes	CHEC Q18
	**Rating for domain**	**GOOD**	0 or 1 = POOR; 2 or 3 = FAIR; 4 = GOOD
**B. Credibility of the clinical trial**	Has the trial generated appropriate and reliable inputs?		
	1. Were participants successfully randomised (i.e. participant characteristics well-matched at baseline)?	Yes	
	2. Are the reported data complete (i.e. no concerns about missing data or participants being lost to follow-up)	Yes	
	3. Were the interventions delivered as intended?	Yes	
	4. Is the duration of the trial appropriate for the inclusion of all relevant costs and consequences?	No	CHEC Q5
	**Rating for domain**	**FAIR**	0 or 1 = POOR; 2 or 3 = FAIR; 4 = GOOD

Domain	Questions and sub-questions	Response	Notes
**C. Credibility of economic evaluation**	Has an appropriate approach been taken for the economic analysis and has the validity of this approach been explored?		
	1. Is the economic study design appropriate to the stated objective?	Yes	CHEC Q4
	2. Is the actual perspective chosen appropriate?	Yes	CHEC Q6
	3. Are all important and relevant costs for each alternative included	Yes	CHEC Q7
	4. Are all costs measured appropriately in physical units?	Yes	CHEC Q8
	5. Are costs valued appropriately?	Yes	CHEC Q9
	6. Is an incremental analysis of costs and outcomes of alternatives performed and clearly reported?	Yes	CHEC Q13
	7. Are all future costs and outcomes discounted appropriately?	No	CHEC Q14
	8. Are all important variables, whose values are uncertain, appropriately subjected to sensitivity analysis?	No	CHEC Q15
	**Rating for domain**	**FAIR**	0 to 3 = POOR; 4 to 7 = FAIR; 8 = GOOD
**D. Certainty of economic evaluation results**	Has the economic analysis generated meaningful outputs?		
	1. Do the conclusions follow from the data reported? Do the authors critically discuss their results including the impact of uncertainty and limitations of the evidence?	Yes	CHEC Q16
	2. Do the authors report any variability measures around their summary estimate of cost-effectiveness (e.g. confidence intervals, a cost-effectiveness acceptability curve)?	Yes	
	3. Does the study discuss the generalizability of the results to other settings and patient/client groups?	Yes	CHEC Q17
	4. Are ethical and distributional issues discussed appropriately?	Yes	CHEC Q19
	**Rating for domain**	**GOOD**	0 or 1 = POOR; 2 or 3 = FAIR; 4 = GOOD

Domain	Questions and sub-questions	Response	Notes
**E. Applicability**<br>Domain	1. Are the sociodemographic characteristics of the population similar to the population of interest, in particular physically inactive individuals?	Yes	
	2. Is the investigated intervention similar to the intervention(s) of interest, in particular, was the intervention aimed at promoting physical activity behaviour change?	Yes	
	3. Are the outcomes investigated appropriate for the review question (i.e. a physical activity outcome, or QOL)?	Yes	
	**Rating for domain**	**Good**	0 or 1 = POOR; 2 = FAIR;<br>3 = GOOD
	Questions and sub-questions	Response	Notes

Level of certainty: MODERATE. The outputs from the economic evaluation are likely to be reliable for decision making, but there is a possibility the outputs are not a reliable prediction of the cost-effectiveness of the intervention

Sevick, 2000

Domain	Questions and sub-questions	Response	Notes
**A. Quality of trial-based economic evaluation reporting**	Is there a clear and comprehensive description of the methods?		
	1. Is the study population clearly described?	Yes	CHEC Q1
	2. Are competing alternatives clearly described?	Yes	CHEC Q2
	3. Is a well-defined research question posed in answerable form?	Yes	CHEC Q3
	4. Does the article indicate that there is no potential conflict of interest of	No	CHEC Q18
	**Rating for domain**	**FAIR**	0 or 1 = POOR; 2 or 3 = FAIR; 4 = GOOD
**B. Credibility of the clinical trial**	Has the trial generated appropriate and reliable inputs?		
	1. Were participants successfully randomised (i.e. participant characteristics well-matched at baseline)?	Yes	
	2. Are the reported data complete (i.e. no concerns about missing data or participants being lost to follow-up)	Yes	
	3. Were the interventions delivered as intended?	Yes	
	4. Is the duration of the trial appropriate for the inclusion of all relevant costs and consequences?	Yes	CHEC Q5
	**Rating for domain**	**GOOD**	0 or 1 = POOR; 2 or 3 = FAIR; 4 = GOOD

Domain	Questions and sub-questions	Response	Notes
**C. Credibility of economic evaluation**	Has an appropriate approach been taken for the economic analysis and has the validity of this approach been explored?		
	1. Is the economic study design appropriate to the stated objective?	Yes	CHEC Q4
	2. Is the actual perspective chosen appropriate?	No	CHEC Q6
	3. Are all important and relevant costs for each alternative included	Yes	CHEC Q7
	4. Are all costs measured appropriately in physical units?	Yes	CHEC Q8
	5. Are costs valued appropriately?	No	CHEC Q9
	6. Is an incremental analysis of costs and outcomes of alternatives performed and clearly reported?	No	CHEC Q13
	7. Are all future costs and outcomes discounted appropriately?	No	CHEC Q14
	8. Are all important variables, whose values are uncertain, appropriately subjected to sensitivity analysis?	No	CHEC Q15
	**Rating for domain**	**FAIR**	0 to 3 = POOR; 4 to 7 = FAIR; 8 = GOOD
**D. Certainty of economic evaluation results**	Has the economic analysis generated meaningful outputs?		
	1. Do the conclusions follow from the data reported? Do the authors critically discuss their results including the impact of uncertainty and limitations of the evidence?	Yes	CHEC Q16
	2. Do the authors report any variability measures around their summary estimate of cost-effectiveness (e.g. confidence intervals, a cost-effectiveness acceptability curve)?	No	
	3. Does the study discuss the generalizability of the results to other settings and patient/client groups?	Yes	CHEC Q17
	4. Are ethical and distributional issues discussed appropriately?	Yes	CHEC Q19
	**Rating for domain**	**FAIR**	0 or 1 = POOR; 2 or 3 = FAIR; 4 = GOOD

Domain	Questions and sub-questions	Response	Notes
**E. Applicability** Domain	1. Are the sociodemographic characteristics of the population similar to the population of interest, in particular physically inactive individuals?	Yes	
	2. Is the investigated intervention similar to the intervention(s) of interest, in particular, was the intervention aimed at promoting physical activity behaviour change?	Yes	
	3. Are the outcomes investigated appropriate for the review question (i.e. a physical activity outcome, or QOL)?	Yes	
	**Rating for domain**	**Good**	0 or 1 = POOR; 2 = FAIR; 3 = GOOD
	Questions and sub-questions	Response	Notes

Level of certainty: MODERATE. The outputs from the economic evaluation are likely to be reliable for decision making, but there is a possibility the outputs are not a reliable prediction of the cost-effectiveness of the intervention

Sevick, 2007

Domain	Questions and sub-questions	Response	Notes
**A. Quality of trial-based economic evaluation reporting**	Is there a clear and comprehensive description of the methods?		
	1. Is the study population clearly described?	Yes	CHEC Q1
	2. Are competing alternatives clearly described?	Yes	CHEC Q2
	3. Is a well-defined research question posed in answerable form?	Yes	CHEC Q3
	4. Does the article indicate that there is no potential conflict of interest of	No	CHEC Q18
	**Rating for domain**	**FAIR**	0 or 1 = POOR; 2 or 3 = FAIR; 4 = GOOD
**B. Credibility of the clinical trial**	Has the trial generated appropriate and reliable inputs?		
	1. Were participants successfully randomised (i.e. participant characteristics well-matched at baseline)?	Yes	
	2. Are the reported data complete (i.e. no concerns about missing data or participants being lost to follow-up)	Yes	
	3. Were the interventions delivered as intended?	Yes	
	4. Is the duration of the trial appropriate for the inclusion of all relevant costs and consequences?	Yes	CHEC Q5
	**Rating for domain**	**GOOD**	0 or 1 = POOR; 2 or 3 = FAIR; 4 = GOOD

Domain	Questions and sub-questions	Response	Notes
**C. Credibility of economic evaluation**	Has an appropriate approach been taken for the economic analysis and has the validity of this approach been explored?		
	1. Is the economic study design appropriate to the stated objective?	Yes	CHEC Q4
	2. Is the actual perspective chosen appropriate?	No	CHEC Q6
	3. Are all important and relevant costs for each alternative included	Yes	CHEC Q7
	4. Are all costs measured appropriately in physical units?	Yes	CHEC Q8
	5. Are costs valued appropriately?	Yes	CHEC Q9
	6. Is an incremental analysis of costs and outcomes of alternatives performed and clearly reported?	No	CHEC Q13
	7. Are all future costs and outcomes discounted appropriately?	Yes	CHEC Q14
	8. Are all important variables, whose values are uncertain, appropriately subjected to sensitivity analysis?	Yes	CHEC Q15
	**Rating for domain**	**FAIR**	0 to 3 = POOR; 4 to 7 = FAIR; 8 = GOOD
**D. Certainty of economic evaluation results**	Has the economic analysis generated meaningful outputs?		
	1. Do the conclusions follow from the data reported? Do the authors critically discuss their results including the impact of uncertainty and limitations of the evidence?	Yes	CHEC Q16
	2. Do the authors report any variability measures around their summary estimate of cost-effectiveness (e.g. confidence intervals, a cost-effectiveness acceptability curve)?	No	
	3. Does the study discuss the generalizability of the results to other settings and patient/client groups?	Yes	CHEC Q17
	4. Are ethical and distributional issues discussed appropriately?	Yes	CHEC Q19
	**Rating for domain**	**FAIR**	0 or 1 = POOR; 2 or 3 = FAIR; 4 = GOOD

Domain	Questions and sub-questions	Response	Notes
**E. Applicability** Domain	1. Are the sociodemographic characteristics of the population similar to the population of interest, in particular physically inactive individuals?	Yes	
	2. Is the investigated intervention similar to the intervention(s) of interest, in particular, was the intervention aimed at promoting physical activity behaviour change?	Yes	
	3. Are the outcomes investigated appropriate for the review question (i.e. a physical activity outcome, or QOL)?	Yes	
	**Rating for domain**	**GOOD**	0 or 1 = POOR; 2 = FAIR; 3 = GOOD
	Questions and sub-questions	Response	Notes

Level of certainty: MODERATE. The outputs from the economic evaluation are likely to be reliable for decision making, but there is a possibility the outputs are not a reliable prediction of the cost-effectiveness of the intervention

Sorensen, 2022

Domain	Questions and sub-questions	Response	Notes
**A. Quality of trial-based economic evaluation reporting**	Is there a clear and comprehensive description of the methods?		
	1. Is the study population clearly described?	Yes	CHEC Q1
	2. Are competing alternatives clearly described?	Yes	CHEC Q2
	3. Is a well-defined research question posed in answerable form?	Yes	CHEC Q3
	4. Does the article indicate that there is no potential conflict of interest of	No	CHEC Q18
	**Rating for domain**	**FAIR**	0 or 1 = POOR; 2 or 3 = FAIR; 4 = GOOD
**B. Credibility of the clinical trial**	Has the trial generated appropriate and reliable inputs?		
	1. Were participants successfully randomised (i.e. participant characteristics well-matched at baseline)?	Yes	
	2. Are the reported data complete (i.e. no concerns about missing data or participants being lost to follow-up)	Yes	
	3. Were the interventions delivered as intended?	Yes	
	4. Is the duration of the trial appropriate for the inclusion of all relevant costs and consequences?	Yes	CHEC Q5
	**Rating for domain**	**GOOD**	0 or 1 = POOR; 2 or 3 = FAIR; 4 = GOOD

Domain	Questions and sub-questions	Response	Notes
**C. Credibility of economic evaluation**	Has an appropriate approach been taken for the economic analysis and has the validity of this approach been explored?		
	1. Is the economic study design appropriate to the stated objective?	Yes	CHEC Q4
	2. Is the actual perspective chosen appropriate?	No	CHEC Q6
	3. Are all important and relevant costs for each alternative included	Yes	CHEC Q7
	4. Are all costs measured appropriately in physical units?	Yes	CHEC Q8
	5. Are costs valued appropriately?	Yes	CHEC Q9
	6. Is an incremental analysis of costs and outcomes of alternatives performed and clearly reported?	No	CHEC Q13
	7. Are all future costs and outcomes discounted appropriately?	Yes	CHEC Q14
	8. Are all important variables, whose values are uncertain, appropriately subjected to sensitivity analysis?	Yes	CHEC Q15
	**Rating for domain**	**FAIR**	0 to 3 = POOR; 4 to 7 = FAIR; 8 = GOOD
**D. Certainty of economic evaluation results**	Has the economic analysis generated meaningful outputs?		
	1. Do the conclusions follow from the data reported? Do the authors critically discuss their results including the impact of uncertainty and limitations of the evidence?	Yes	CHEC Q16
	2. Do the authors report any variability measures around their summary estimate of cost-effectiveness (e.g. confidence intervals, a cost-effectiveness acceptability curve)?	Yes	
	3. Does the study discuss the generalizability of the results to other settings and patient/client groups?	Yes	CHEC Q17
	4. Are ethical and distributional issues discussed appropriately?	Yes	CHEC Q19
	**Rating for domain**	**GOOD**	0 or 1 = POOR; 2 or 3 = FAIR; 4 = GOOD

Domain	Questions and sub-questions	Response	Notes
**E. Applicability**<br>Domain	1. Are the sociodemographic characteristics of the population similar to the population of interest, in particular physically inactive individuals?	No	
	2. Is the investigated intervention similar to the intervention(s) of interest, in particular, was the intervention aimed at promoting physical activity behaviour change?	Yes	
	3. Are the outcomes investigated appropriate for the review question (i.e. a physical activity outcome, or QOL)?	Yes	
	**Rating for domain**	**FAIR**	0 or 1 = POOR; 2 = FAIR;<br>3 = GOOD
	Questions and sub-questions	Response	Notes

Level of certainty: MODERATE. The outputs from the economic evaluation are likely to be reliable for decision making, but there is a possibility the outputs are not a reliable prediction of the cost-effectiveness of the intervention

Turkstra, 2013

Domain	Questions and sub-questions	Response	Notes
**A. Quality of trial-based economic evaluation reporting**	Is there a clear and comprehensive description of the methods?		
	1. Is the study population clearly described?	Yes	CHEC Q1
	2. Are competing alternatives clearly described?	Yes	CHEC Q2
	3. Is a well-defined research question posed in answerable form?	Yes	CHEC Q3
	4. Does the article indicate that there is no potential conflict of interest of	No	CHEC Q18
	**Rating for domain**	**FAIR**	0 or 1 = POOR; 2 or 3 = FAIR; 4 = GOOD
**B. Credibility of the clinical trial**	Has the trial generated appropriate and reliable inputs?		
	1. Were participants successfully randomised (i.e. participant characteristics well-matched at baseline)?	Yes	
	2. Are the reported data complete (i.e. no concerns about missing data or participants being lost to follow-up)	Yes	
	3. Were the interventions delivered as intended?	Yes	
	4. Is the duration of the trial appropriate for the inclusion of all relevant costs and consequences?	Yes	CHEC Q5
	**Rating for domain**	**GOOD**	0 or 1 = POOR; 2 or 3 = FAIR; 4 = GOOD

Domain	Questions and sub-questions	Response	Notes
**C. Credibility of economic evaluation**	Has an appropriate approach been taken for the economic analysis and has the validity of this approach been explored?		
	1. Is the economic study design appropriate to the stated objective?	Yes	CHEC Q4
	2. Is the actual perspective chosen appropriate?	No	CHEC Q6
	3. Are all important and relevant costs for each alternative included	No	CHEC Q7
	4. Are all costs measured appropriately in physical units?	Yes	CHEC Q8
	5. Are costs valued appropriately?	Yes	CHEC Q9
	6. Is an incremental analysis of costs and outcomes of alternatives performed and clearly reported?	Yes	CHEC Q13
	7. Are all future costs and outcomes discounted appropriately?	No	CHEC Q14
	8. Are all important variables, whose values are uncertain, appropriately subjected to sensitivity analysis?	No	CHEC Q15
	**Rating for domain**	**FAIR**	0 to 3 = POOR; 4 to 7 = FAIR; 8 = GOOD
**D. Certainty of economic evaluation results**	Has the economic analysis generated meaningful outputs?		
	1. Do the conclusions follow from the data reported? Do the authors critically discuss their results including the impact of uncertainty and limitations of the evidence?	Yes	CHEC Q16
	2. Do the authors report any variability measures around their summary estimate of cost-effectiveness (e.g. confidence intervals, a cost-effectiveness acceptability curve)?	No	
	3. Does the study discuss the generalizability of the results to other settings and patient/client groups?	No	CHEC Q17
	4. Are ethical and distributional issues discussed appropriately?	Yes	CHEC Q19
	**Rating for domain**	**FAIR**	0 or 1 = POOR; 2 or 3 = FAIR; 4 = GOOD

Domain	Questions and sub-questions	Response	Notes
**E. Applicability** Domain	1. Are the sociodemographic characteristics of the population similar to the population of interest, in particular physically inactive individuals?	Yes	
	2. Is the investigated intervention similar to the intervention(s) of interest, in particular, was the intervention aimed at promoting physical activity behaviour change?	Yes	
	3. Are the outcomes investigated appropriate for the review question (i.e. a physical activity outcome, or QOL)?	Yes	
	**Rating for domain**	**GOOD**	0 or 1 = POOR; 2 = FAIR; 3 = GOOD
	Questions and sub-questions	Response	Notes

Level of certainty: MODERATE. The outputs from the economic evaluation are likely to be reliable for decision making, but there is a possibility the outputs are not a reliable prediction of the cost-effectiveness of the intervention

**Level of certainty**	**Definition**	**How it is derived**
HIGH	We are confident that the outputs from the economic evaluation are reliable for decision-making	All domains in Table 1 are rated Good
MODERATE	The outputs from the economic evaluation are likely to be reliable for decision making, but there is a possibility the outputs are not a reliable prediction of the cost-effectiveness of the intervention	All domains in Table 1 are rated Fair or higher
LOW	We have limited confidence that the outputs from the economic evaluation are reliable for decision-making	One domain in Table 1 is rated Poor but all other domains are rated Fair or Good
VERY LOW	We have very little confidence that the outputs from the economic evaluation are reliable for decision-making	More than one domain in Table 1 is rated Poor

vanKeulen, 2010

Domain	Questions and sub-questions	Response	Notes
**A. Quality of trial-based economic evaluation reporting**	Is there a clear and comprehensive description of the methods?		
	1. Is the study population clearly described?	Yes	CHEC Q1
	2. Are competing alternatives clearly described?	Yes	CHEC Q2
	3. Is a well-defined research question posed in answerable form?	Yes	CHEC Q3
	4. Does the article indicate that there is no potential conflict of interest of	No	CHEC Q18
	**Rating for domain**	**FAIR**	0 or 1 = POOR; 2 or 3 = FAIR; 4 = GOOD
**B. Credibility of the clinical trial**	Has the trial generated appropriate and reliable inputs?		
	1. Were participants successfully randomised (i.e. participant characteristics well-matched at baseline)?	Yes	
	2. Are the reported data complete (i.e. no concerns about missing data or participants being lost to follow-up)	No	
	3. Were the interventions delivered as intended?	Yes	
	4. Is the duration of the trial appropriate for the inclusion of all relevant costs and consequences?	Yes	CHEC Q5
	**Rating for domain**	**FAIR**	0 or 1 = POOR; 2 or 3 = FAIR; 4 = GOOD

Domain	Questions and sub-questions	Response	Notes
**C. Credibility of economic evaluation**	Has an appropriate approach been taken for the economic analysis and has the validity of this approach been explored?		
	1. Is the economic study design appropriate to the stated objective?	Yes	CHEC Q4
	2. Is the actual perspective chosen appropriate?	No	CHEC Q6
	3. Are all important and relevant costs for each alternative included	Yes	CHEC Q7
	4. Are all costs measured appropriately in physical units?	Yes	CHEC Q8
	5. Are costs valued appropriately?	Yes	CHEC Q9
	6. Is an incremental analysis of costs and outcomes of alternatives performed and clearly reported?	Yes	CHEC Q13
	7. Are all future costs and outcomes discounted appropriately?	No	CHEC Q14
	8. Are all important variables, whose values are uncertain, appropriately subjected to sensitivity analysis?	No	CHEC Q15
	**Rating for domain**	**FAIR**	0 to 3 = POOR; 4 to 7 = FAIR; 8 = GOOD
**D. Certainty of economic evaluation results**	Has the economic analysis generated meaningful outputs?		
	1. Do the conclusions follow from the data reported? Do the authors critically discuss their results including the impact of uncertainty and limitations of the evidence?	Yes	CHEC Q16
	2. Do the authors report any variability measures around their summary estimate of cost-effectiveness (e.g. confidence intervals, a cost-effectiveness acceptability curve)?	Yes	
	3. Does the study discuss the generalizability of the results to other settings and patient/client groups?	Yes	CHEC Q17
	4. Are ethical and distributional issues discussed appropriately?	Yes	CHEC Q19
	**Rating for domain**	**GOOD**	0 or 1 = POOR; 2 or 3 = FAIR; 4 = GOOD

Domain	Questions and sub-questions	Response	Notes
**E. Applicability**<br>Domain	1. Are the sociodemographic characteristics of the population similar to the population of interest, in particular physically inactive individuals?	No	
	2. Is the investigated intervention similar to the intervention(s) of interest, in particular, was the intervention aimed at promoting physical activity behaviour change?	Yes	
	3. Are the outcomes investigated appropriate for the review question (i.e. a physical activity outcome, or QOL)?	Yes	
	**Rating for domain**	**FAIR**	0 or 1 = POOR; 2 = FAIR;<br>3 = GOOD
	Questions and sub-questions	Response	Notes

Level of certainty: MODERATE. The outputs from the economic evaluation are likely to be reliable for decision making, but there is a possibility the outputs are not a reliable prediction of the cost-effectiveness of the intervention

Additional file 5. Records excluded at full-text screening and reasons for exclusion

Authors	Published Year	Title	Journal	Reason
Babazono, Akira; Kame, Chihoko; Ishihara, Reiko; Yamamoto, Eiji; Hillman, Alan L. et al	2007	Patient-motivated prevention of lifestyle-related disease in Japan: A randomized, controlled clinical trial	Disease Management & Health Outcomes	Wrong study design
Brodin, Nina; Karlsson, Malin Lohela; Swardh, Emma; Opava, Christina H.	2013	Cost-Effectiveness and Cost-Utility Of a One-Year Coaching Program For Healthy Physical Activity In Rheumatoid Arthritis	Arthritis and Rheumatism	Wrong Outcomes
Brouwers, Rutger W. M.; van der Poort, Esmee K. J.; Kemps, Hareld M. C.; van den Akker-van Marle, M. Elske; Kraal, Jos J.	2021	Cost-effectiveness of Cardiac Telerehabilitation With Relapse Prevention for the Treatment of Patients With Coronary Artery Disease in the Netherlands	JAMA Network Open	Wrong intervention
Canaway, Alastair; Pincus, Tamar; Underwood, Martin; Shapiro, Yair; Chodick, Gabriel; Ben-Ami, Noa	2018	Is an enhanced behaviour change intervention cost-effective compared with physiotherapy for patients with chronic low back pain? Results from a multicentre trial in Israel	BMJ Open	Wrong Outcomes
Cobiac, Linda; Vos, Theo; Veerman, Lennert	2010	Cost-effectiveness of Weight Watchers and the Lighten Up to a Healthy Lifestyle program	Australian and New Zealand Journal of Public Health	Wrong Outcomes
de Vries, Nienke M.; Staal, J. Bart; van der Wees, Philip J.; Adang, Eddy M. M.; Akkermans, Reinier; Rikkert, Marcel G. M. Olde; Nijhuis-van der Sanden, Maria W. G.	2016	Patient-centred physical therapy is (cost-) effective in increasing physical activity and reducing frailty in older adults with mobility problems: a randomized controlled trial with 6months follow-up	JOURNAL OF CACHEXIA SARCOPENIA AND MUSCLE	Wrong intervention
Deidda, Manuela; Coll-Planas, Laura; Tully, Mark A.; Gine-Garriga, Maria; Kee, Frank; Figuls, Marta	2022	Cost-effectiveness of a programme to address sedentary behaviour in older adults: results from the SITLESS RCT	European Journal of Public Health	Wrong intervention

Roque I.; Blackburn, Nicole E.; Guerra-Balic, Miriam; Rothenbacher, Dietrich; Dallmeier, Dhayana; Caserotti, Paolo; Skjodt, Mathias; McIntosh, Emma; Sitless Consortium				
Dwinger, Sarah; Rezvani, Farhad; Kriston, Levente; Herbarth, Lutz; Haerter, Martin; Dirmaier, Joerg	2020	Effects of telephone-based health coaching on patient-reported outcomes and health behavior change: A randomized controlled trial	PLoS ONE	Wrong study design
Dzator, J. A.; Hendrie, D.; Burke, V.; Gianguilio, N.; Gillam, H. F.; Beilin, L. J.; Houghton, S.	2004	A randomized trial of interactive group sessions achieved greater improvements in nutrition and physical activity at a tiny increase in cost	Journal of Clinical Epidemiology	Wrong Outcomes
Emmons, Karen M.; Puleo, Elaine; Greaney, Mary L.; Gillman, Matthew W.; Bennett, Gary G.; Haines, Jess; Sprunck-Harrild, Kim; Viswanath, K.	2014	A randomized comparative effectiveness study of Healthy Directions 2-A multiple risk behavior intervention for primary care	Preventive Medicine	Wrong Outcomes
Eriksson, M. K.; Hagberg, L.; Lindholm, L.; Malmgren-Olsson, E. B.; Osterlind, J.; Eliasson, M.	2010	Quality of Life and Cost-effectiveness of a 3-Year Trial of Lifestyle Intervention in Primary Health Care	Archives of Internal Medicine	Wrong Outcomes
Eriksson, M. K.; Malmgren-Olsson, E. B.; Hagberg, L. A.; Eliasson, M.	2010	Lifestyle intervention, quality of life and cost-effectiveness, a randomized controlled trial	European Journal of Cardiovascular Prevention and Rehabilitation	Supplementary article
Ewald, Ben; Stacey, Fiona; Johnson, Natalie; Plotnikoff, Ronald C.; Holliday, Elizabeth; Brown, Wendy; James, Erica L.	2018	Physical activity coaching by Australian Exercise Physiologists is cost effective for patients referred from general practice	Australian and New Zealand Journal of Public Health	No cost-effectiveness analysis
Gordon, L. G.; Patrao, T.; Kularatna, S.; Hawkes, A. L.	2015	A telephone-delivered multiple health behaviour change intervention for colorectal cancer survivors: making the case for cost-effective healthcare	European journal of cancer care	Wrong Outcomes

Groessl, E. J.; Kaplan, R. M.; Blair, S. N.; Rejeski, W. J.; Katula, J. A.; King, A. C.; Fielding, R. A.; Glynn, N. W.; Pahor, M.	2009	A cost analysis of a physical activity intervention for older adults	Journal of Physical Activity and Health	Wrong intervention
Gyllensten, Hanna; Haby, Karin; Berg, Marie; Premberg, Asa	2021	Cost effectiveness of a controlled lifestyle intervention for pregnant women with obesity	BMC Pregnancy and Childbirth	Wrong Outcomes
Hind, D.; Scott, E. J.; Copeland, R.; Breckon, J. D.; Crank, H.; Walters, S. J.; Brazier, J. E.; Nicholl, J.; Cooper, C.; Goyder, E.	2010	A randomised controlled trial and cost-effectiveness evaluation of "booster" interventions to sustain increases in physical activity in middle-aged adults in deprived urban neighbourhoods	BMC public health	Wrong intervention
Hinman, Rana S.; Campbell, Penny K.; Lawford, Belinda J.; Briggs, Andrew M.; Gale, Janette; Bills, Caroline; Kasza, Jessica; Harris, Anthony; French, Simon D.; Bunker, Stephen J.; Forbes, Andrew; Bennell, Kim L.	2020	Does telephone-delivered exercise advice and support by physiotherapists improve pain and/or function in people with knee osteoarthritis? Telecare randomised controlled trial	British Journal of Sports Medicine	Wrong intervention
Hunter, R. F.; Murray, J. M.; Gough, A.; Tang, J.; Patterson, C. C.; French, D. P.; McIntosh, E.; Xin, Y.; Kee, F.	2018	Effectiveness and cost-effectiveness of a loyalty scheme for physical activity behaviour change maintenance: Results from a cluster randomised controlled trial	International Journal of Behavioral Nutrition and Physical Activity	Wrong study design
Kempf, K., Röhling, M., Stichert, M., Fischer, G., Boschem, E., Könner, J., & Martin, S.	2018	Telemedical coaching improves long-term weight loss in overweight persons: a randomized controlled trial	nternational journal of telemedicine and applications	No cost-effectiveness analysis
Kivela, Kirsi; Elo, Satu; Kyngas, Helvi; Kaariainen, Maria	2020	The effects of nurse -led health coaching on health -related quality of life and clinical health outcomes among frequent attenders: A quasi -experimental study	Patient Education and Counseling	Wrong study design

Kruger, J.; Brennan, A.; Strong, M.; Thomas, C.; Norman, P.; Epton, T.	2014	The cost-effectiveness of a theory-based online health behaviour intervention for new university students: an economic evaluation	BMC public health	Wrong study design
Larsen, B.; Marcus, B.; Pekmezi, D.; Hartman, S.; Gilmer, T.	2017	A Web-Based Physical Activity Intervention for Spanish-Speaking Latinas: A Costs and Cost-Effectiveness Analysis	Journal of Medical Internet Research	Wrong intervention
Looijmans, A.; Jorg, F.; Bruggeman, R.; Schoevers, R.; Corpeleijn, E.	2017	Design of the Lifestyle Interventions for severe mentally ill Outpatients in the Netherlands (LION) trial; a cluster randomised controlled study of a multidimensional web tool intervention to improve cardiometabolic health in patients with severe mental illness	BMC Psychiatry	Wrong study design
Macfarlane, G. J.; Beasley, M.; Scott, N.; Chong, H.; McNamee, P.; McBeth, J.; Basu, N.; Hannaford, P. C.; Jones, G. T.; Keeley, P.; Prescott, G. J.; Lovell, K.	2021	Maintaining musculoskeletal health using a behavioural therapy approach: A population-based randomised controlled trial (the MAmMOTH Study)	Annals of the Rheumatic Diseases	Wrong intervention
Maddison, Ralph; Pfaeffli, Leila; Whittaker, Robyn; Stewart, Ralph; Kerr, Andrew; Jiang, Yannan; Kira, Geoffrey; Leung, William; Dalleck, Lance; Carter, Karen; Rawstorn, Jonathan	2015	A mobile phone intervention increases physical activity in people with cardiovascular disease: Results from the HEART randomized controlled trial	European Journal of Preventive Cardiology	Wrong intervention
Maple, Jaimie-Lee; Ananthapavan, Jaithri; Ball, Kylie; Teychenne, Megan; Moodie, Marj	2022	Economic evaluation of an incentive-based program to increase physical activity and reduce sedentary behaviour in middle-aged adults	BMC health services research	Wrong study design
McConnon, A.; Kirk, S. F. L.; Cockroft, J. E.; Harvey, E. L.; Greenwood, D. C.; Thomas, J. D.; Ransley, J. K.; Bojke, L.	2007	The Internet for weight control in an obese sample: Results of a randomised controlled trial	BMC health services research	Wrong intervention

Murphy, Simon Mark; Edwards, Rhiannon Tudor; Williams, Nefyn; Raisanen, Larry; Moore, Graham; Linck, Pat; Hounsome, Natalia; Din, Nafees Ud; Moore, Laurence	2012	An evaluation of the effectiveness and cost effectiveness of the National Exercise Referral Scheme in Wales, UK: a randomised controlled trial of a public health policy initiative	Journal of Epidemiology and Community Health	Wrong intervention
Neumann, Anne; Lindholm, Lars; Norberg, Margareta; Schoffer, Olaf; Klug, Stefanie J.; Norstrom, Fredrik	2017	The Cost-Effectiveness of Interventions Targeting Lifestyle Change for the Prevention of Diabetes in a Swedish Primary Care and Community Based Prevention Program	European Journal of Health Economics	Wrong intervention
Rom, Ã…Sa; Persson, Ulf; Ekdahl, Charlotte; Gard, Gunvor	2014	Costs and outcomes of an exercise referral programme - A 1-year follow-up study	European Journal of Physiotherapy	Wrong intervention
Rome, Asa; Persson, Ulf; Ekdahl, Charlotte; Gard, Gunvor	2009	Physical activity on prescription (PAP): Costs and consequences of a randomized, controlled trial in primary healthcare	Scandinavian Journal of Primary Health Care	Wrong study design
Rothberg, Amy E.; McEwen, Laura N.; Kraftson, Andrew T.; Neshewat, Gina M.; Fowler, Christine E.; Burant, Charles F.; Herman, William H.	2014	The impact of weight loss on health-related quality-of-life: implications for cost-effectiveness analyses	Quality of Life Research	Wrong study design
Saha, S.; Lindgren, P.; Johansson, P.; Gerdtham, U. G.	2010	Economic evaluation of lifestyle intervention in primary prevention of cardiovascular disease in 60-year-old men in Stockholm, Sweden	Value in Health	Wrong study design
Sangster, Janice; Furber, Susan; Phongsavan, Philayrath; Redfern, Julie; Mark, Andrew; Bauman, Adrian	2017	Effects of a Pedometer-Based Telephone Coaching Intervention on Physical Activity Among People with Cardiac Disease in Urban, Rural and Semi-Rural Settings: A Replication Study	Heart Lung and Circulation	Wrong study design
Sanz-Guinea, A.; Espinosa, M.; Grandes, G.; Sancheza, A.; Martinez, C.; Pombo, H.; Bully, P.; Cortada, J.	2017	Efficiency of "Prescribe Vida Saludable", a health promotion innovation. Pilot phase	Gaceta sanitaria	Wrong intervention

Schulz, D. N.; Smit, E. S.; Stanczyk, N. E.; Kremers, S. P.; de Vries, H.; Evers, S. M.	2014	Economic evaluation of a web-based tailored lifestyle intervention for adults: findings regarding cost-effectiveness and cost-utility from a randomized controlled trial	Journal of Medical Internet Research	Wrong intervention
Schulz, Daniela N.; Smit, Eline S.; Stanczyk, Nicola E.; Kremers, Stef P. J.; de Vries, Hein et al.	2014	Economic evaluation of a Web-based tailored lifestyle intervention for adults: Findings regarding cost-effectiveness and cost-utility from a randomized a controlled trial	Journal of Medical Internet Research	Wrong intervention
Sevick, M. A.; Miller, G. D.; Loeser, R. F.; Williamson, J. D.; Messier, S. P.	2009	Cost-effectiveness of exercise and diet in overweight and obese adults with knee osteoarthritis	Medicine and science in sports and exercise	Wrong intervention
Simpson, Sharon A.; McNamara, Rachel; Shaw, Christine; Kelson, Mark; Moriarty, Yvonne; Randell, Elizabeth et al.	2015	A feasibility randomised controlled trial of a motivational interviewing-based intervention for weight loss maintenance in adults	Health Technology Assessment	Wrong intervention
Smith, K. C.; Paltiel, A. D.; Yang, H. Y.; Collins, J. E.; Katz, J. N.; Losina, E.	2018	Cost-effectiveness of health coaching and financial incentives to promote physical activity after total knee replacement	Osteoarthritis and Cartilage	Wrong study design
Stanmore, Emma K.; Mavroeidi, Alexandra; de Jong, Lex D.; Skelton, Dawn A.; Sutton, Chris J.; Benedetto, Valerio; Munford, Luke A.; Meekes, Wytske; Bell, Vicky; Todd, Chris	2019	The effectiveness and cost-effectiveness of strength and balance Exergames to reduce falls risk for people aged 55years and older in UK assisted living facilities: a multi-centre, cluster randomised controlled trial	BMC Medicine	Wrong intervention
Turkstra, E.; Hawkes, A. L.; Oldenburg, B. F.; Scuffham, P. A.	2012	Cost-effectiveness of a secondary prevention program in patients with myocardial infarction: Results from a randomised controlled trial (proactive heart)	Value in Health	Supplementary article
Ussher, Michael; Lewis, Sarah; Aveyard, Paul; Manyonda, Isaac; West, Robert; Lewis, Beth;	2015	The London Exercise And Pregnant smokers (LEAP) trial: a randomised controlled trial of physical activity for	Health Technology Assessment	Wrong intervention

Marcus, Bess; Riaz, Muhammad; Taylor, Adrian H.; Barton, Pelham; Daley, Amanda; Essex, Holly; Esliger, Dale; Coleman, Tim		smoking cessation in pregnancy with an economic evaluation		
van Beers, Martijn; Rutten-van Molken, Maureen P. M. H.; van de Bool, Coby; Boland, Melinde; Kremers et al	2020	Clinical outcome and cost-effectiveness of a 1-year nutritional intervention programme in COPD patients with low muscle mass: The randomized controlled NUTRAIN trial	Clinical Nutrition	Wrong intervention
van Dongen, E. J. I.; Haveman-Nies, A.; Wezenbeek, N. L. W.; Dorhout, B. G.; Doets, E. L.; de Groot, L. C. P. G. M.	2018	Effect, process, and economic evaluation of a combined resistance exercise and diet intervention (ProMuscle in Practice) for community-dwelling older adults: design and methods of a randomised controlled trial	BMC public health	Wrong study design
Verhaeghe, N.; De Smedt, D.; De Maeseneer, J.; Maes, L.; Van Heeringen, C.; Annemans, L.	2014	Cost-effectiveness of health promotion targeting physical activity and healthy eating in mental health care	BMC public health	Wrong intervention
Wang, Hua; Kenkel, Donald; Graham, Meredith L.; Paul, Lynn C.; Folta, Sara C.; Nelson, Miriam E.; Strogatz, David; Seguin, Rebecca A.	2019	Cost-effectiveness of a community-based cardiovascular disease prevention intervention in medically underserved rural areas	BMC health services research	Wrong intervention
Whelan, M. E.; Goode, A. D.; Eakin, E. G.; Veerman, J. L.; Winkler, E. A. H.; Hickman, I. J.; Reeves, M. M.	2016	Feasibility, effectiveness and cost-effectiveness of a telephone-based weight loss program delivered via a hospital outpatient setting	Translational Behavioral Medicine	Wrong intervention
Wyke, Sally; Bunn, Christopher; Andersen, Eivind; Silva, Marlene N.; van Nassau, Femke; McSkimming, Paula et al.	2019	The effect of a programme to improve men's sedentary time and physical activity: The European Fans in Training (EuroFIT) randomised controlled trial	PLoS Medicine	Wrong intervention
Wylie-Rosett, Judith; Herman, William H.; Goldberg, Ronald B.	2006	Lifestyle intervention to prevent diabetes: intensive AND cost effective	Current Opinion in Lipidology	Wrong study design

Additional file 6. Risk of bias assessment of economic evaluations included in the review using the Consensus on Health Economic Criteria list (CHEC-list)

	**CHEC questions and answers**																			
**Study, year**	**1**	**2**	**3**	**4**	**5**	**6**	**7**	**8**	**9**	**10**	**11**	**12**	**13**	**14**	**15**	**16**	**17**	**18**	**19**	**Certainty of evidence**
Barrett, 2019	Yes	Yes	Yes	Yes	No	No	No	Yes	Yes	Yes	Yes	Yes	Yes	Yes	Yes	No	Yes	No	No	Moderate
Barrett, 2022	Yes	Yes	Yes	Yes	Yes	No	Yes	Yes	No	Yes	Yes	Yes	Yes	No	Yes	Yes	Yes	Yes	Yes	Moderate
Brodin, 2015	Yes	No	Yes	Yes	No	Yes	Yes	Yes	Yes	Yes	Yes	Yes	Yes	No	Yes	Yes	Yes	Yes	No	Moderate
Broekhuizen, 2018	Yes	No	Yes	Yes	Yes	Yes	Yes	Yes	Yes	Yes	Yes	Yes	Yes	No	Yes	Yes	No	Yes	No	Moderate
Buder, 2018	Yes	Yes	Yes	No	Yes	No	Yes	Yes	No	Yes	No	Yes	No	No	Yes	Yes	Yes	No	No	Low
Crist et al, 2022	Yes	No	Yes	Yes	Yes	No	Yes	Yes	No	No	Yes	Yes	Yes	No	No	Yes	Yes	Yes	Yes	Moderate
Goyder, 2014	Yes	Yes	Yes	Yes	Yes	Yes	Yes	Yes	Yes	Yes	Yes	Yes	Yes	Yes	Yes	Yes	Yes	Yes	Yes	High
Ismail, 2020	Yes	Yes	Yes	Yes	Yes	No	Yes	No	Yes	Yes	Yes	Yes	Yes	No	Yes	Yes	Yes	No	Yes	Moderate
Jacobs, 2010	Yes	Yes	Yes	Yes	Yes	No	Yes	Yes	Yes	Yes	Yes	Yes	Yes	Yes	Yes	Yes	Yes	no	Yes	Moderate
Khunti, 2021	Yes	Yes	Yes	Yes	Yes	Yes	Yes	Yes	Yes	Yes	Yes	Yes	Yes	Yes	Yes	Yes	Yes	No	Yes	Moderate
Sangster, 2015	Yes	Yes	Yes	Yes	No	Yes	Yes	Yes	Yes	Yes	Yes	Yes	Yes	No	No	Yes	Yes	Yes	Yes	Moderate
Sevick, 2000	Yes	Yes	Yes	Yes	Yes	No	Yes	Yes	No	Yes	Yes	Yes	No	Yes	Yes	Yes	Yes	No	Yes	Moderate
Sevick, 2007	Yes	Yes	Yes	Yes	Yes	No	Yes	Yes	Yes	Yes	Yes	Yes	No	Yes	Yes	Yes	Yes	No	Yes	Moderate
Sorensen, 2022	Yes	No	Yes	Yes	Yes	No	Yes	Yes	No	Yes	Yes	Yes	Yes	Yes	No	Yes	Yes	Yes	Yes	Moderate
Turkstra, 2013	Yes	Yes	Yes	Yes	Yes	No	No	Yes	Yes	Yes	Yes	Yes	Yes	No	No	Yes	No	No	Yes	Moderate
vanKeulen, 2010	Yes	Yes	Yes	Yes	Yes	No	Yes	Yes	Yes	Yes	Yes	Yes	Yes	No	No	Yes	Yes	No	Yes	Moderate

1. Is the study population clearly described? 2. Are competing alternatives clearly described? 3. Is a well-defined research question posed in answerable form? 4. Is the economic study design appropriate to the stated objective? 5. Is the chosen time horizon appropriate in order to include relevant costs and consequences? 6. Is the actual perspective chosen appropriate? 7. Are all important and relevant costs for each alternative identified? 8. Are all costs measured appropriately in physical units? 9. Are costs valued appropriately? 10. Are all important and relevant outcomes for each alternative identified? 11. Are all outcomes measured appropriately? 12. Are outcomes valued appropriately? 13. Is an incremental analysis of costs and outcomes of alternatives performed? 14. Are all future costs and outcomes discounted appropriately? 15. Are all important variables, whose values are uncertain, appropriately subjected to sensitivity analysis? 16. Do the conclusions follow from the data reported? 17. Does the study discuss the generalizability of the results to other settings and patient/client groups? 18. Does the article indicate that there is no potential conflict of interest of study researcher(s) and funder(s)? 19. Are ethical and distributional issues discussed appropriately?

Level of certainty: High: We are confident that the outputs from the economic evaluation are reliable for decision-making. Moderate: The outputs from the economic evaluation are likely to be reliable for decision making, but there is a possibility the outputs are not a reliable prediction of the cost-effectiveness of the intervention. Low: We have limited confidence that the outputs from the economic evaluation are reliable for decision-making. Very low: We have very little confidence that the outputs from the economic evaluation are reliable for decision-making.
